# Molecular breeding approaches for sustainable rice blast management: recent advances and challenges

**DOI:** 10.3389/fpls.2025.1551018

**Published:** 2025-09-23

**Authors:** Sravanthi Ragulakollu, Arul Loganathan, Manonmani Swaminatham, Gopalakrishnan Chellappan, Ravichandran Veeraswamy, Ramalingam Jegadeesan

**Affiliations:** ^1^ Department of Genetics and Plant Breeding, Tamil Nadu Agricultural University, Coimbatore, Tamil Nadu, India; ^2^ Department of Plant Biotechnology, Centre for Plant Molecular Biology and Biotechnology, Tamil Nadu Agricultural University, Coimbatore, Tamil Nadu, India; ^3^ Department of Rice, Tamil Nadu Agricultural University, Coimbatore, Tamil Nadu, India; ^4^ Department of Plant Pathology, Tamil Nadu Agricultural University, Coimbatore, Tamil Nadu, India; ^5^ Department of Crop Physiology, Tamil Nadu Agricultural University, Coimbatore, Tamil Nadu, India

**Keywords:** rice, blast disease, MAS, MABB, CRISPR/Cas9, omics approaches, nanotechnology, artificial intelligence

## Abstract

Rice (*Oryza sativa*. L) is a staple crop globally, but blast disease caused by fungal pathogens *Magnaporthe oryzae* is one of the most devastating and results in severe economic losses in rice production worldwide. Recent technological advancements have opened new possibilities for developing blast resistance. The dynamic and highly adaptable nature of *M. oryzae* allows it to overcome plant defense mechanisms rapidly, posing a major threat to global food security and agricultural sustainability. While foundational to early resistance development, traditional breeding approaches have been limited by their time-consuming nature and reliance on phenotypic selection. These methods often require several generations to establish stable resistance traits. However, with the emergence of molecular breeding technologies, resistance breeding has experienced significant acceleration and precision. Tools such as marker-assisted selection (MAS), marker-assisted backcross breeding (MABB), and quantitative trait locus (QTL) mapping allow for the identification and introgression of resistance genes (R genes) more efficiently and accurately. Recent advances in genome engineering techniques, particularly CRISPR-Cas 9, have transformed the capability to manipulate resistance genes directly, enabling targeted editing and stacking of multiple genes (gene pyramiding) for durable resistance. Moreover, omics technologies—including genomics, transcriptomics, proteomics, and metabolomics—offer a comprehensive understanding of the molecular interactions between host and pathogen, facilitating the discovery of novel resistance mechanisms and regulatory pathways. The integration of allele mining with advanced biotechnological tools has further promoted the development of cisgenic and intragenic plants, where resistance genes from related cultivars or wild species are introduced without foreign DNA, thus addressing public concerns over transgenic crops. These strategies enhance resistance and help retain the desirable agronomic traits of elite rice varieties. Despite these advancements, the high mutation rate and genetic plasticity of *M. oryzae* enable it to evolve and overcome resistance provided by single R genes. Therefore, understanding host–pathogen interactions at the molecular and cellular levels remains essential. Emerging technologies such as nanotechnology show promise in developing targeted fungicide delivery systems and innovative diagnostic tools. Synthetic biology opens avenues for constructing synthetic resistance pathways or deploying plant biosensors. Additionally, machine learning and artificial intelligence (AI) algorithms are increasingly used to predict disease outbreaks, model gene interactions, and optimize breeding strategies based on large datasets. Thus, managing rice blast disease necessitates a holistic approach combining conventional breeding wisdom with modern molecular tools and emerging technologies. The synergy among these approaches holds promise to enhance resistance durability and protect global rice production against evolving fungal threats. This review emphasizes recent advancements in managing rice blast disease, offering valuable insights to sustain resilient breeding programs against this pathogen.

## Graphical Abstract

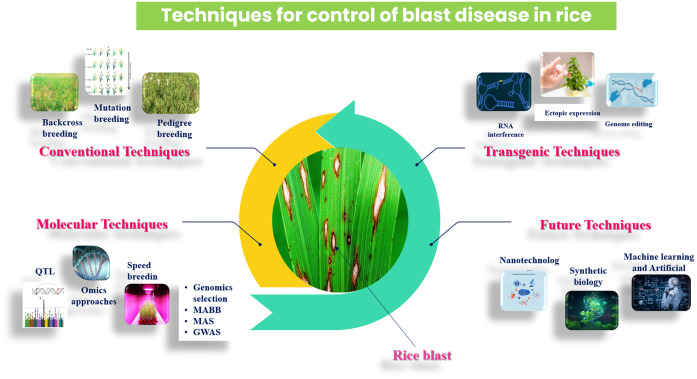

## Introduction

Rice (*Oryza sativa* L.) is considered the most crucial staple food crop, sustaining over half of the global population (Maurya et al., 2024). Biotic and abiotic stresses significantly impact yield losses in food production, thus necessitating the improvement of stress tolerance in crops. Among these stresses, disease stands out as a primary limiting stress factor in rice crop production (Zeng et al., 2023). Rice is susceptible to more than 70 diseases caused by various fungi, nematodes, viruses, and bacteria (Rijal and Devkota, 2020). Rice blast, caused by *Magnaporthe oryzae* (Anamorph: *Pyricularia oryzae*), is a particularly widespread and notorious fungal pathogen that can reduce grain yield and quality by 70%–80% (Simkhada and Thapa, 2022). This disease affects all parts of the rice plants and can lead to complete yield loss under favorable conditions worldwide. Leaf blast and panicle blast are two economically significant forms of disease (Ding et al., 1999). Leaf blast impairs photosynthesis and reduces carbohydrate production (Bastiaans, 1993), typically resulting in 1% to 10% yield losses (Savary et al., 2000); severe epidemics can cause leaf death or kill entire plants in early stages (Ou, 1985). Panicle blast, which is generally more economically impactful, can diminish yield by hindering grain filling, especially near the panicle base, potentially leading to the loss of the entire panicle (Zeigler et al., 1994). Approximately 30% of the yield loss is attributed to collar and neck blasts caused by spores produced later in the growth season (Srivastava et al., 2017). Climate change may alter pathogen distribution and growth rates and affect host plant resistance, growth, and metabolism. Lower temperatures in humid tropics and warm, humid subtropical regions increase the risk of blast epidemics (Ninomiya et al., 2020).

The management of rice blast disease primarily relies on traditional breeding techniques, such as pedigree, backcross, mutation, and recurrent selection, which often face challenges with linkage drag, where undesirable traits are inadvertently transferred along with resistance genes. These conventional methods have limitations in effectively controlling blast disease and are associated with high labor costs and a time-consuming process. Consequently, researchers have employed molecular markers to identify blast resistance using various techniques, including QTL mapping, genetic transformation, and marker-aided selection (MAS). Integrating marker-assisted selection (MAS) with traditional breeding methods has enabled the accumulation of R genes in elite rice cultivars, enhancing their resistance to blast and improving durability. These approaches involve enhanced selection processes that focus on improved quality and desirable traits, leading to the development of blast-resistant rice varieties (Ashkani et al., 2015). Thus, understanding the molecular interactions between the pathogen and the host plant is essential to develop effective strategies to manage and control rice blast disease. Additionally, recent studies have identified specific blast resistance genes, offering new opportunities for breeding programs (Ramkumar et al., 2015; Amoghavarsha et al., 2022). This review briefly examines conventional methods and molecular approaches such as transgenic techniques, CRISPR/Cas9 technology, nanotechnology, allele mining, and omics strategies that contribute to the management of rice blast disease, offering insights into its control.

## Blast in rice

*Magnaporthe oryzae* and *Magnaporthe grisea* are closely related fungal pathogens known to cause rice blast disease. However, *M. oryzae* is considered the primary and more aggressive pathogen responsible for rice blast, whereas *M. grisea* is generally associated with other grasses and is less effective in infecting rice (Choi et al., 2013). Their pathogenicity and host specificity differ significantly.

### Host specificity and pathogenicity

*Magnaporthe oryzae*: Primarily responsible for rice blast disease, *M. oryzae* infects a broad range of grasses, including economically important crops such as wheat, barley, millet, and maize. Its adaptability to various hosts makes it a significant threat to global cereal production (Chung et al., 2020).

*Magnaporthe grisea*: Initially isolated from crabgrass (*Digitaria sanguinalis*), *M. grisea* exhibits a more restricted host range, predominantly infecting species within the *Digitaria* genus (Zhang et al., 2015).

#### Comparative effects on rice blast disease

**Table:** 

Feature	*Magnaporthe oryzae*	*Magnaporthe grisea*
Primary host	Rice (*Oryza sativa*)	Grasses (e.g., *Setaria*, *Digitaria*)
Pathogenicity	Highly virulent in rice	Less virulent in rice
Genetic specialization	Adapted to rice with specific host–pathogen interactions	More diverse host range but less aggressive on rice
Effector proteins	Contains *Avr* genes (e.g., *Avr-Pita*, *Avr-Pik*) that interact with rice resistance genes	Lacks some key *Avr* genes specific to rice
Disease severity	Causes severe yield losses, up to 30%–50% in epidemic conditions	Causes minimal damage to rice crops
Global impact	Most devastating fungal disease of rice	Not a major pathogen of rice

In summary, *Magnaporthe oryzae* is more adept at causing rice blast disease than *Magnaporthe grisea*. This conclusion is supported by recent studies highlighting the broader host range and higher pathogenicity of *M. oryzae* in rice and other cereal crops.

### Pathogen description and diversity

The presence of multiple races within a blast pathogen intensifies its interaction with the host, overpowering the host’s defense system. A total of 25 cultivars, *viz*., recombinant inbred lines, commercial cultivars, and donors, are generally used to monitor the virulence of blast pathogens, according to the All India Coordinated Rice Improvement Programme (Hasan et al., 2024). This pathogen is highly variable and rapidly evolves into novel pathotypes. In the 1970s, the identification of a novel race group took place—IJ, which emerged from Indian isolates of *M. oryzae*, with the IC3 and ID1 races standing out prominently (Padmanabhan et al., 1970). Notably, ID-17 was prevalent within Indian paddy ecosystems among five pathogenic race groups—ID-1, ID-2, IB-4, IC-17, and IC-25. Evaluation of the genetic heterogeneity of *Magnaporthe* species in both rice and finger millet ecosystems in southern India involved the utilization of simple sequence repeats (SSRs) and repetitive DNA-based markers targeting pathogenicity genes (Palanna et al., 2024). To establish a durable system to protect against blast disease since 2006, the Japan International Research Center for Agricultural Sciences (JIRCAS) has been conducting a collaborative study, “Blast Research Network for Stable Rice Production,” targeting Southeast and East Asia. According to the gene for gene hypothesis, the complex interaction occurs between host resistance and fungus virulence in rice blast pathogens; every resistance gene in the host corresponds to an avirulence gene in the pathogen. Based on this theory, differential varieties (DVs), which can be used to distinguish pathotypes (races) by their reaction patterns to each pathogen strain, have been developed to identify the blast pathogen population structure and predict the emergence of new blast races. Using several sets of DVs, pathogenicity studies of blast isolates have been performed in China and Southeast Asia. Using 12 Japanese differential varieties (DVs) for *Pia*, *Pik-s*, *Pii*, *Pik*, *Pik-m*, *Piz*, *Pita*, *Pita-2*, *Piz-t*, *Pik-p*, *Pib*, and *Pit* (Kiyosawa, 1984; Yamada et al., 1976; Tsunematsu et al., 2000), 12 kinds of blast race have been identified among 129 isolates collected from all over the Mekong River Delta area of Vietnam. A total of 25 monogenic lines harboring 23 resistance genes, namely, *Pish*, *Pib*, *Pit*, *Pia*, *Pii*, *Pi3*, *Pi5*(t), *Pik-s*, *Pik-m*, *Pi1*, *Pik-h*, *Pik*, *Pik-p*, *Pi7*(t), *Pi9(t)*, *Piz*, *Piz-5*, *Piz-t*, *Pita-2*, *Pita*, *Pi12*(t), *Pi19*(t), and *Pi20*(t), were developed as a new set of international DVs by several backcrosses using the Chinese susceptible rice variety LTH and a set of LTH NILs carrying 11 resistance genes (*Pib*, *Piz-5*, *Pi9(t)*, *Pi3*, *Pia*, *Pik-s*, *Pik*, *Pik-h*, *Pi7*(t), *Pita*, and *Pita-2*) developed by Telebanco-Yanoria et al. (2010). However, there has been no research into blast races in Cambodia, nor has any information on blast disease or genotypes of rice varieties been collected. The pathogenicity of blast isolates was based on inoculation test using differential varieties in near-isogenic lines in Japan (Kawasaki-Tanaka et al., 2016), Laos (Xangsayasane et al., 2020), Vietnam (Fukuta et al., 2020; Nguyet et al., 2020), Indonesia, Bangladesh (Khan et al., 2016), West Africa (Odjo et al., 2014), and Kenya (Fukuta et al., 2019). These DVs had the common genetic background of LTH (Lijiangxintuanheigu), meaning that the influence of genetic background on the appearance of blast symptoms was minimized. The targets of the DV sets and other useful materials released are major blast resistance genes, which have been used internationally as part of JIRCAS’s research. These monogenic lines and LTH NILs are being used to develop differential systems in each country through (1) pathogenicity analysis of blast isolates, (2) elucidation of blast race distribution, and (3) selection of standard differential blast isolates. In the context of *Magnaporthe oryzae* interactions with rice differentials in the CO39 background, several virulence and avirulence (*AVR*) genes that play crucial roles in determining the pathogen’s specificity and the host’s resistance response have been identified. Near-isogenic lines (NILs) were developed to study the interaction between rice and *Magnaporthe oryzae* by introgressing individual resistance (*R*) genes from diverse donor parents while maintaining a uniform genetic background. CO39, an *Indica* rice variety, was selected as the recurrent parent due to its high susceptibility to blast disease, allowing a clear evaluation of the effects of individual *R* genes. Each NIL carries a single *R* gene introduced from different donor parents through successive backcrossing and marker-assisted selection. NILs are composed of 14 R genes: *Pish*, *Pib*, *Piz-5*, *Piz-t*, *Pi5(t)*, *Pik-s*, *Pik*, *Pik-h*, *Pik-m*, *Pik-p*, *Pi1*, *Pi7(t)*, *Pita*, and *Pita-2*, each derived from different donor varieties such as Toride 1, Fukunishiki, Kanto 51, K60, Tetep, and Tsuyuake. Telebanco-Yanoria et al. (2011) developed blast-resistant rice varieties through multiple backcrossing cycles and selecting specific *M. oryzae* isolates to confirm the presence of the targeted R genes. Further phenotypic evaluations showed that these NILs displayed reaction patterns akin to monogenic lines containing identical R genes when tested against standard Philippine isolates. This resemblance highlights their significance as differential varieties in studying blast resistance, especially in areas where Japonica-type varieties such as LTH are less impactful (Yang et al., 2022). Within the framework of CO39 rice cultivar, *AVR1-CO39* has been studied as a significant locus that regulates the extensive avirulence of *M. oryzae* strain 2539 on cultivated rice. This gene aligns with the resistance gene *Pi-CO39(t)* in rice, where it has been genetically mapped to a specific locus on the short arm of chromosome 11 and has shown effectiveness against various isolates, highlighting its evolutionary importance (Zheng et al., 2011). Consequently, it can be inferred that *AVR1-CO39* is specific to the species rather than the cultivar, functioning as a host-specific *AVR* locus for *M. oryzae* in rice (Tosa et al., 2005). Notably, both *AVR1-CO39* and *AVR-Pia* are recognized by the rice resistance proteins RGA4 and RGA5, which collaboratively initiate immune responses (Petit-Houdenot and Fudal, 2017). Additionally, the AVR-*Pik* locus exhibits high haplotype diversity, with novel variants evolved through stepwise base substitutions, enabling the pathogen to overcome *Pik*-mediated resistance (Li J et al., 2019). Unraveling the intricacies of *AVR* gene functions is crucial to develop effective strategies to combat blast disease resistance in rice.

### Geographic distribution

**Figure 1:**
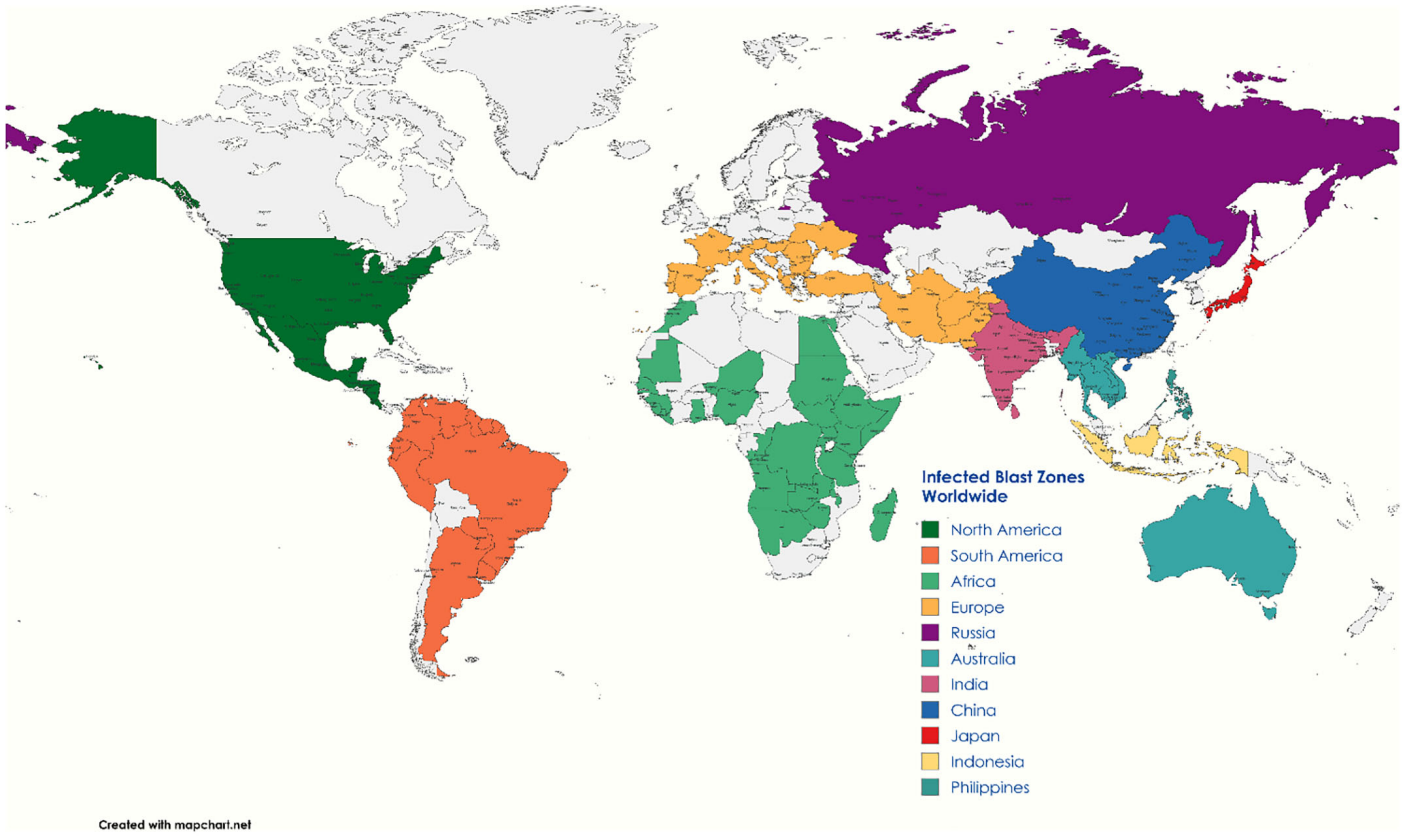
Geographical distribution of *Magnaporthe oryzae* in rice.

Rice production in West Africa, the world’s largest rice producer, is severely affected by blast disease, causing yield losses of 3%–77% (Shahriar et al., 2020). This disease, also referred to as rice seedling blight (Zeigler et al., 1994) and rice rotten neck (Talbot, 2003), was first documented as “rice fever” in China in 1637 and later as Imochi-byo in Japan in 1704. It gained global recognition with Italy experiencing its first epidemic in 1828 and the Tanjore delta in India identifying it in 1919. Currently, the disease impacts approximately 85 countries worldwide, particularly in South Asia and Africa (Wang et al., 2017), with annual yield losses ranging from 10% to 80% (Simkhada and Thapa, 2022). These losses are influenced by various factors such as varietal susceptibility, infection severity, fungicide application timing, high humidity, drought, heavy dew, elevated mean temperatures, high plant density, and excessive nitrogen fertilizer use. In India, rice blast epidemics can result in yield losses of up to 50%. During natural epidemics in the wet season, disease incidence ranges from 14% to 27% (exceeding the economic threshold), leading to yield losses of about 27%–35%. Severe epidemics occurred between 1980 and 1987 in several Indian states, such as Himachal Pradesh, Andhra Pradesh, Tamil Nadu, and Haryana, causing substantial financial losses. It is estimated that the annual yield reduction due to rice blast disease could feed approximately 60 million people each year. In the United States, Arkansas, Louisiana, and Mississippi are the most affected states, with yield losses ranging from 6% to 50% and an average annual loss of USD 69.34 million due to blast. The disease is also a significant concern in European countries like Italy, Spain, Portugal, Greece, and France, where it has been observed to reduce the milling yield by 20% to 50% (Devanna et al., 2022) (**Figure 1**).

### Interaction between host plant and blast pathogen in rice

**Figure 2:**
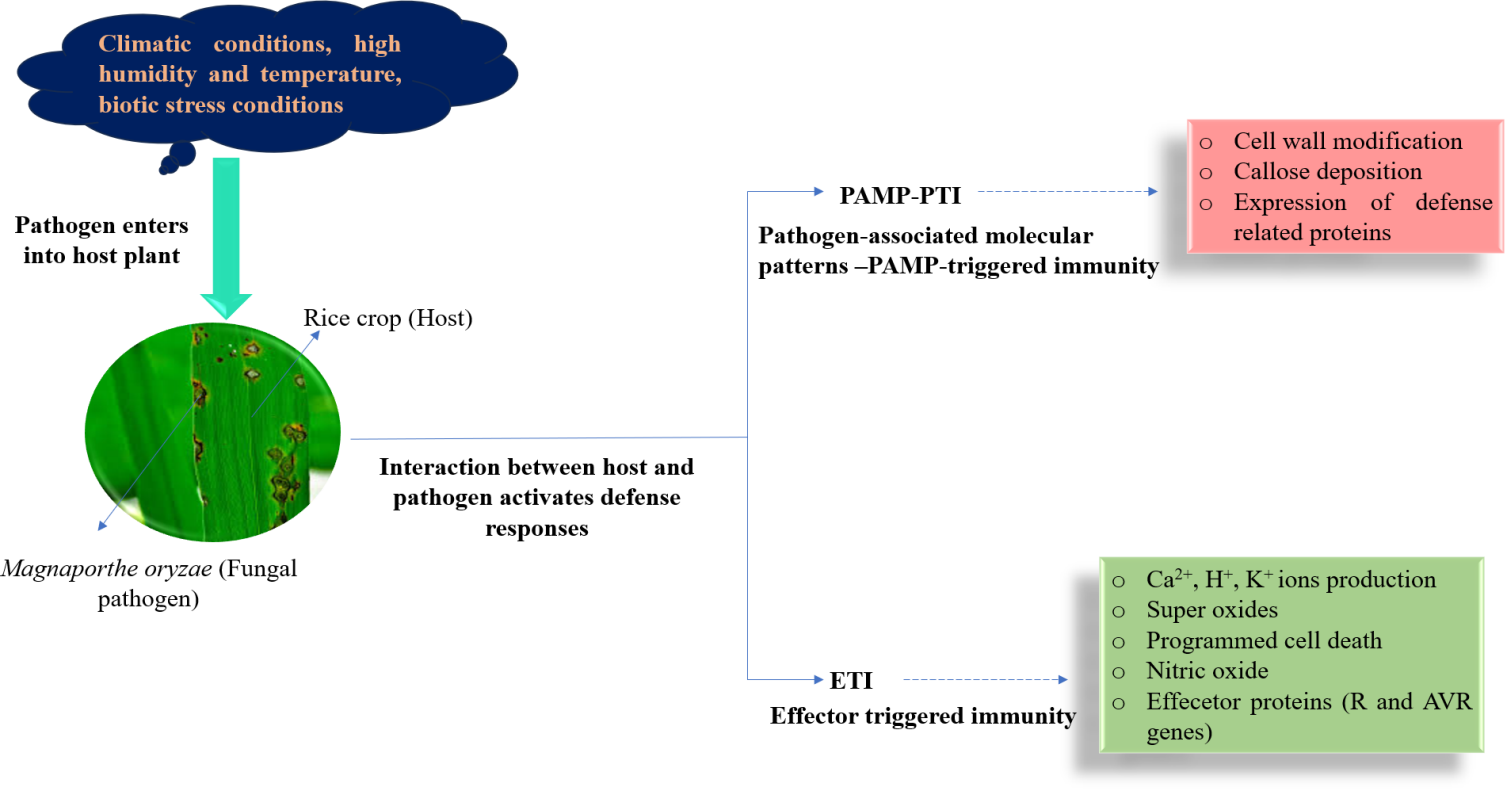
Different defense mechanisms occur when the interaction between *Magnaporthe oryzae* and rice.

Once the rice is infected by *M. oryzae*, pattern recognition receptors (PRRs) on the cell surface can specifically recognize pathogen-associated molecule patterns (PAMPs) and activate defense response by cell wall modification, callose deposition, and *via* the expression of defense-related proteins in host cells, which is termed PAMP-triggered immunity (PTI) (**Figure 2**). However, PTI is a weak and non-specific resistance mechanism (Bernoux et al., 2011). In many cases, *M. oryzae* can secrete certain effectors to inhibit PAMP-induced PTI and break resistance responses (Jones and Dangl, 2006; Mentlak et al., 2012). At the same time, rice has acquired more specific resistance proteins that directly or indirectly recognize pathogen-effector proteins. This recognition mechanism activates a second layer of the defense response in rice, known as effector-triggered immunity (ETI), which results in the production of ion (Ca^2+^, K^+^, and H^+^) currents, superoxide, nitric oxide, and programmed cell death at the site of invasion (Nurnberger et al., 2004). ETI is a highly specialized disease resistance mechanism in the host (Boller and He, 2009), which is activated in the gene-for-gene model upon recognition by an R (resistance) protein of the corresponding effector protein of *M. oryzae*. Effector proteins are often encoded by avirulence genes in *M. oryzae*. The R genes in rice correspond to the avirulence (*AVR*) genes in *M. oryzae* in a gene-for-gene manner (Flor, 1971), which ensures that the interaction between a specific R protein in rice and the corresponding AVR effector in the pathogen renders resistance. The R protein encoded by R genes interacts directly or indirectly with the effector protein, thus sensing pathogen invasion and inducing disease resistance. Despite the deployment of resistant varieties, blast epidemics can still occur due to a lapse in host resistance and the emergence of new virulent pathotypes (Chuma et al., 2011). Thus, the effectiveness of R genes depends on the respective *AVR* gene.

### Various mechanisms underlying *Magnaporthe oryzae* and rice plant

Rice plants have developed complex defense mechanisms to recognize and combat the rice blast fungus, *Magnaporthe oryzae*. Understanding these mechanisms is crucial to develop effective breeding methods aimed at producing disease-resistant rice varieties (Saklani et al., 2023). In their interaction with *M. oryzae*, rice plants utilize pattern recognition receptors (PRRs) to detect pathogen-associated molecular patterns (PAMPs). This detection triggers PAMP-triggered immunity (PTI), which starts early defensive responses—for instance, when rice PRRs identify chitin fragments from the fungal cell wall, it activates signaling pathways that promote the generation of reactive oxygen species (ROS) and the expression of defense-related genes (Zhang L et al., 2024).

### Pattern-triggered immunity

Plants possess sophisticated immune systems that can identify and respond to pathogenic threats through two connected mechanisms: pattern-triggered immunity (PTI) and Effector-Triggered Immunity (ETI). PTI activates when pattern recognition receptors (PRRs) on the surface of plant cells detect conserved pathogen-associated molecular patterns (PAMPs), such as bacterial flagellin or fungal chitin. This detection triggers a series of defensive actions, including the production of reactive oxygen species, reinforcement of the cell wall, and the activation of defense-related genes, collectively impeding pathogen invasion. To bypass PTI, pathogens excrete effector proteins that can interfere with these defenses. In response, plants have developed intracellular nucleotide-binding leucine-rich repeat (NB-LRR) proteins that can detect these effectors, which leads to ETI. ETI generally manifests as a hypersensitive response, characterized by localized cell death at the infection site, limiting the pathogens’ spread. The dynamic interaction between PTI and ETI forms a robust defense network that enables plants to recognize various pathogens and initiate appropriate immune responses (Dodds et al., 2024). In rice, the initial defense mechanism relies on pattern recognition receptors (PRRs) on the plasma membrane to identify pathogen-associated molecular patterns (PAMPs). A notable PRR in rice is the chitin elicitor binding protein (CEBiP), which specifically binds to chitin fragments from fungal cell walls. Upon recognizing chitin, CEBiP associates with another receptor-like kinase, CERK1, which activates a signaling cascade that leads to PAMP-triggered immunity (PTI). This immune response includes the generation of reactive oxygen species (ROS), activation of mitogen-activated protein kinases (MAPKs), and the expression of defense-related genes, all contributing to the prevention of pathogen invasion (Mentlak et al., 2012).

### Effector-triggered immunity

To inhibit PTI, *Magnaporthe oryzae* secretes effector proteins into the host cells. Rice plants have evolved resistance (R) genes that encode nucleotide-binding leucine-rich repeat (NLR) proteins that specifically detect these effectors. This recognition activates effector-triggered immunity (ETI), a strong defense mechanism that often leads to localized cell death, referred to as the hypersensitive response (HR), to thwart the pathogen (Tian et al., 2018). Effector-triggered immunity (ETI) plays a crucial role in plant defense, enabling the identification of specific pathogen effectors, particularly in the interaction between rice (*Oryza sativa*) and *Magnaporthe oryzae*. In this gene-for-gene model, rice plants contain resistance (R) genes coding for intracellular NLR proteins that recognize corresponding avirulence (AVR) effectors released by *M. oryzae*. This interaction triggers a vigorous immune response, often resulting in localized cell death to manage the pathogen, known as the hypersensitive response. A notable instance is the relationship between the rice R gene *Piz-t* and the *M. oryzae* AVR effector *AvrPiz-t* (Liu X et al., 2024). The *AvrPiz-t* gene encodes a secreted protein that when identified by the *Piz-t* protein in rice, initiates ETI, effectively stopping disease development. However, *M. oryzae* can escape this defense by modifying, deleting, or inserting transposons into its AVR genes, leading to altered effectors that the plant’s R proteins cannot recognize. This ongoing evolutionary struggle necessitates continuous monitoring of AVR gene changes and the creation of rice cultivars with diverse durable R genes to manage blast disease effectively (Zhang and Xu, 2014). Reactive oxygen species (ROS) play a critical role in PTI and ETI, serving as signaling molecules and direct agents against pathogens. In PTI, ROS are generated in a swift burst that strengthens cell walls and activates defense genes. In ETI, ROS accumulation is prolonged, contributing to the hypersensitive response, reinforcing cell walls, and triggering programmed cell death to control the pathogen.

**Figure 3:**
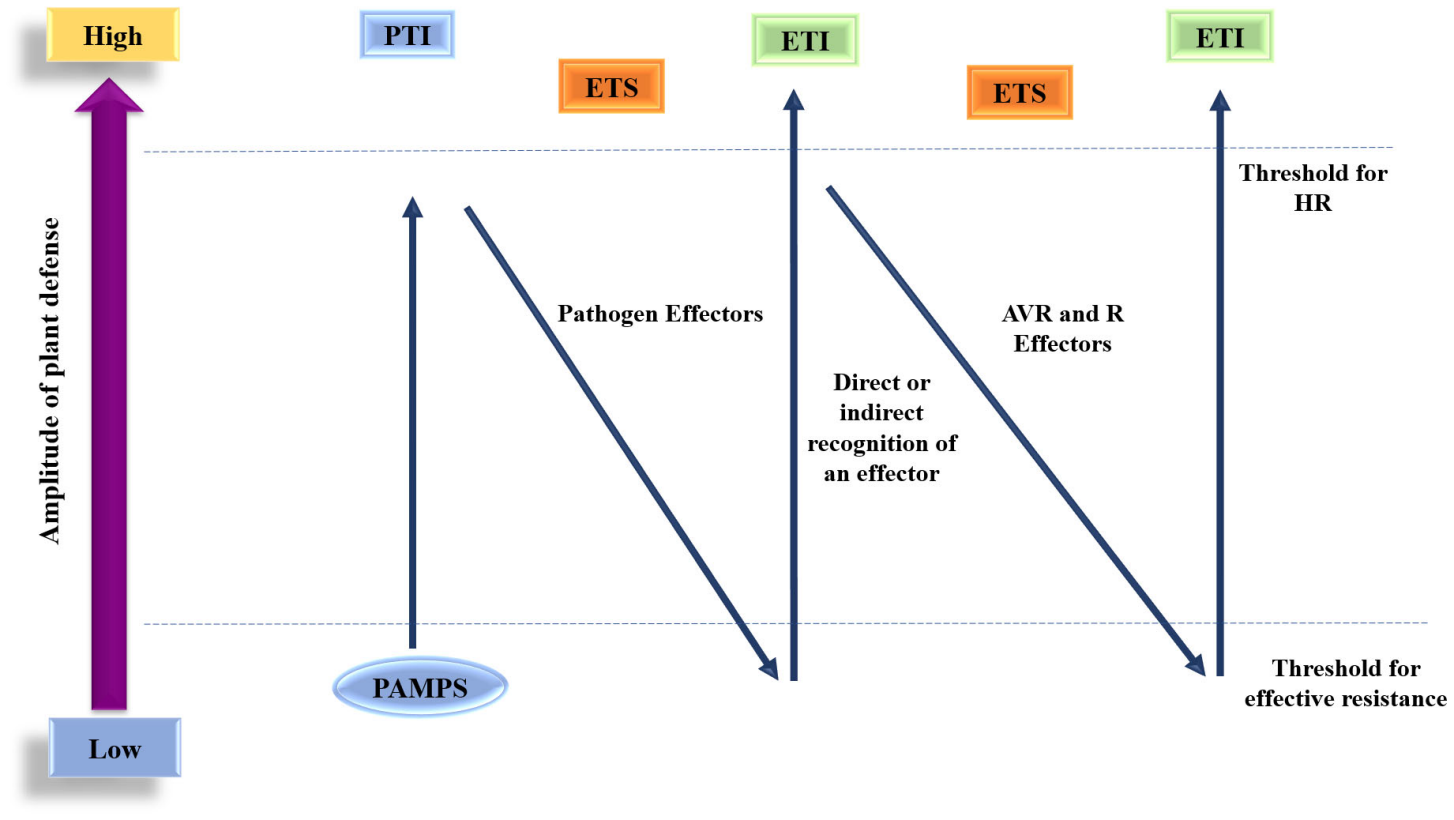
Zig-zag manner for disease mechanism between *M. oryzae* and rice.

The zig-zag model, initially proposed by Jones and Dangl in 2006, describes how the recognition of PAMPs initiates primary defense mechanisms based on PTI, which help to reduce pathogen growth, though they do not completely eliminate it. Pathogens that flourish have developed effector or virulence factors that promote their growth by suppressing PTI, resulting in effector-triggered susceptibility (ETS). In response to certain pathogen effectors, plants have developed ETI, primarily by recognizing altered self-products from ETS *via* NB-LRR. This continuous evolutionary battle between host and pathogen manifests in repeating cycles of ETS and subsequent ETI. The outcome of the plant-pathogen interaction is influenced by the sum of ([PTI − ETS] + ETI) (**Figure 3**).

Understanding these molecular interactions provides essential insights for breeding strategies. By identifying and integrating R genes that can detect a broad spectrum of *M. oryzae* effectors, breeders can develop rice varieties with enduring resistance. Additionally, enhancing the expression of PRRs like CEBiP or other components involved in ROS production may strengthen basic immunity, creating a multi-tiered defense against the pathogen. Breeding approaches that incorporate multiple resistance genes, coupled with understanding the dynamic interplay between rice and *M. oryzae*, can enhance durable resistance. Future research should focus on integrating these molecular insights into advanced breeding techniques such as CRISPR-Cas9 genome editing to achieve broad-spectrum and long-lasting resistance in rice.

### Interaction of host R genes with AVR genes

To date, 26 *Avr*/effector genes have been mapped in *M. oryzae*, and 14 of them, including two unmapped *Avrs*, *MoHTR1* and *MoHTR2*, have been cloned and characterized (**Table 1**). The first discovered *Pwl* effectors (*Pwl*1–*Pwl*4) belong to a small, glycine-rich, rapidly evolving effector family that provides avirulence on weeping lovegrass and finger millet but does not affect rice. Except for cell death induction/suppression or interaction with resistance protein features, identifying candidate effector proteins is a difficult task due to their unique sequence features. Among the 26 reported *Avr*-genes, 15 were mapped near the chromosome ends, and five of the cloned *Avr* genes were flanked by transposons. These transposons are active companions of the *Avr* genes and play a role in the loss and gain of these genes. The molecular interaction studies of the reported seven *R-Avr* pairs showed that five of them, namely, *Pi-ta*/*AVR-Pita*, *Pik/AVR-Pik*, *Pia/AVR-Pia*, *Pi-CO39/AVR1-CO39*, and *Pi54/AVR Pi54* interact directly, whereas *Piz-t/AvrPiz-t* and *Pii/AVR-Pii* have indirect interaction.

**Table 1:** List of cloned avirulence genes.

Effector type	Cognate R gene	*AVR* genes	Protein size	Chromosome	References
ToxB like	*Pi54, Pi54rh, Pi54of*	*AVR-Pi54*	153	4	Ray et al., 2016
	*Pia*	*AVR-Pita*	85	5/7	Orbach et al., 2000
	*Pik/Pik-m/Pik-p, Pik-h*	*AVR-Pik/km/kp; (AVR-Pikh)*	113	1	Yoshida et al., 2009; Wu et al., 2014
	*Piz-t*	*AvrPiz-t*	108	7	Li et al., 2009
Zinc metalloprotease	*Pita*	*AVR-Pita*	224	3	Orbach et al., 2000
PKS/NRPS	*Pi33* (not cloned)	*ACE1*	4,035	1	Bohnert et al., 2004
Six cysteine	*Pi9(t)*	*AVR-Pi9(t)*	91	7	Wu Y et al., 2015
Zinc-finger TF	Unknown	*MoHTR1*	Unknown	Unknown	Kim et al., 2020
Glycine rich	Unknown	*PWL1*	147	2	Kang et al., 1995
		*PWL2*	145	2	Sweigard et al., 1995
Unknown	*Pii*	*AVR-Pii*	70	7	Yoshida et al., 2009
	*Pib*	*AVR-Pib*	75	3	Zhang et al., 2015

### Common R–AVR pairs and their role against rice blast resistance

#### 1. *Pi-ta* and *AVR-Pita*

*AVR-Pita* and *Pi-ta* from the fungal pathogen *M. oryzae* are among the first R–Avr interactions to be thoroughly explored (Wang et al., 2019). This interaction has played a pivotal role in establishing a fundamental understanding of the complex dynamics between plants and pathogens, specifically about disease initiation and developing resistance mechanisms. The gene *AVR-Pita*, which is located near to telomeres and is responsible for encoding a protein, secretes and possesses a unique domain known as Zn metalloprotease (Khang et al., 2008). The *Avr-Pita* protein attains its mature state as a protease, consisting of a sequence of 176 amino acids located at the C-terminus (Razzaq et al., 2019). *Avr*-Pita is a member of a unique subclass within the *AVR-Pita* gene family, comprising three different genes: *AVR-Pita1*, *AVR-Pita2*, and *AVR-Pita3*. The first two genes mentioned possess functional properties that initiate Pi-ta-mediated resistance, whereas the third gene is a pseudogene lacking *Avr f*unctionality (Ribaut, 2006). The *Pi-ta* R gene counterpart is a conventional NLR (928 amino acids) receptor situated in the cytoplasm and generally exhibits constitutive expression (Wu J et al., 2015). The direct interaction between the leucine-rich domain (LRD) of the *Pi-ta* protein and the *AVR Pita*176 protein leads to the activation of downstream signaling cascades. The utilization of site-directed mutagenesis has facilitated the functional validation of *AVR*-Pita, leading to the identification of two critical amino acid substitutions, *AVR*-pita176E177D and *AVR* pita176M178W, which result in the loss of its virulence function. In a similar vein, the presence of a mutated form of the *Pi-ta* R gene, characterized by a single amino acid substitution (LRDA918S), has been observed to reduce the physical interaction between the *AVR-Pita*176 and *Pi-ta* LRD proteins. This finding underscores the significance of the interplay between *R–Avr* pairs in the establishment of immunity against *M. oryzae* (Hossain et al., 2018).

#### 2. *Pia* and *AVR-Pia*

This is the second class of interaction in which two NLRs, RGA4 and RGA5, interact with a single *Avr* protein (Mutiga et al., 2021; Wang et al., 2019). The encoded secretory protein of *AVR-Pia* contains an N-terminal SP (Martin et al., 2003). Different isolates of *M. oryzae* that are resistant to *Pia* genes in rice have different numbers of copies, ranging from one to three; this depends on the isolate. For instance, the avirulent strain Ina168 possesses three copies of *AVR-Pia* genes (Ribot et al., 2013). The NMR (nuclear magnetic resonance)- determined structure of *AVR-Pia* reveals a MAX effector β-sandwich-like structure, while *Pia* is composed of RGA4 and RGA5 protein genes, oriented face-to-face in opposite directions (Mutiga et al., 2021). Furthermore, two isoforms of RGA5 called RGA5-A and RGA5-B are the consequences of RGA5 alternative splicing, in which only RGA5-A mediates Pia resistance. In *in vitro* experiments, it has been observed that the continuous production of RGA4 leads to the initiation of cell death. However, in the absence of infection, this cell death is suppressed by RGA5 in planta. It is important to note that the NB (nucleotide-binding) domain of RGA4 is essential for the induction of cell death (Fukoka et al., 2009; Hayashi K et al., 2010). Physical contact between *AVR-Pia* and the non-LRR C-terminal domain of RGA5 facilitates the inhibition and promotion of RGA4-mediated cell damage.

#### 3. *Pii* and *AVR-Pii*

This is the third type of interaction in which the *R–Avr* pair (*Pii* and *AVR-Pii*) mediates the immune response through an indirect interaction (Martin et al., 2003). The secreted protein encoded by *AVR-Pii* belongs to a protein family known as pex33. The protein structure comprises four homologs with two conserved motifs (Yoshida et al., 2009). Conversely, the protein encoded by *Pii* is a common NLR consisting of 1025 amino acids (Mutiga et al., 2021). Two forms (I and II) of *AVR-Pii* exist in different isolates. Form I is a hybrid of rice proteins (OsExo70-F2 and OsExo70-F3) and *AVR-Pii*. Though both rice proteins are required for the immune response, the latter (OsExo70-F3) instead of the former rice protein, induces a *Pii*-mediated immune response. This result implies that OsExo70 serves as a helper protein in the interaction of *Pii/AVR-Pii* (Hossain et al., 2018).

#### 4. *Piz-t* and *AVR-Piz-t*

One such form of *R–AVR* contact pertains to the indirect interaction that occurs between *Piz-t* and *AVR Piz-t*, which is a classic example of a plant–pathogen interaction where a single, broad-spectrum R gene recognizes and interacts with multiple variants of an *AVR* gene (Liu et al., 2014). The *AVR* Piz-t protein has secretory characteristics akin to those of other well-known *AVR* genes. The structure of *AVR Piz-t* and similar *ToXB* genes was determined *via* NMR. *AVR* Piz-t comprises a β-sheet consisting of six disulfide chains from Cys62 to Cys75. Single point mutations on any cysteine residue reduce the toxicity of *AVR Piz-t* (Sone et al., 2013). *Piz-t* functions as a broad-spectrum NLR gene. The LRR domain of *Piz-t* exhibits 18 amino acid alterations, which determine the activation of resistance and differentiate *Piz-t* from *Pi-2* (Zhou et al., 2006). Being a broad-spectrum R gene, twelve different interacting proteins of *AVR Piz-t* (APIPs) interact with *AVR Piz-t* in various lines of rice. The nature of resistance or immunological response is contingent upon the specific *AVR Piz-t* protein and the genetic composition of the rice host harboring the *Piz-t gene*. In the context of *Piz-t-*lacking Nippon bare rice, the suppression of PTI is observed as a result of the interaction between *AVR Piz-t* and PTI. Conversely, in the presence of *Piz-t*, PTI is stabilized when the rice plant is infected by *M. oryzae* (Fujisaki et al., 2015).

### Cloning of blast resistance genes in rice

Analysis of rice germplasm with different races reveals complete resistance conferred by significant blast resistance (R) gene. However, the resistance may be broken down due to its single R gene locus having race-specific characteristics. Because of the progress in molecular marker development and functional genomics, blast resistance genetics in rice have been strengthened (Qian et al., 2023). R genes serve as the cornerstone of disease resistance. To date, 146 resistance genes of the blast have been identified; among them, 41 of these genes (*Pib*, *Pit*, *Pish*, *Pita*, *Pi54*, *Pi-d2*, *Ptr*, *Pi9(t)*, *Pia*, *Pi- C039*, *Pi65*, *Pi2*, *Piz-t*, *Piz-h*, *Pig-m*, *Pi50*, *Pi36*, *Pi37*, *Pik-h*, *Pik-m*, *Pi1*, *Pik-e*, *Pi56*, *Pi-d3*, *Pi25*, *pi21*, *Pb1*, *Pi5(t)*, *Pii*, *Pik*, *Pid3A4*, *Pi35*, *PiPR1*, *Pi64*, *Pik-p*, *Pi63*, *Pid3-11*, *Pb2*, *Ptr*, and *Pi54-rh*) have been cloned and functionally authenticated (Sahu et al., 2022; Younas et al., 2023). Among the array of identified resistance genes against blast disease, *Pi54* and *Pi2* are highly effective and provide broad-spectrum resistance (Aleena et al., 2023). The genetic analysis, mapping, and cloning of rice blast resistance have been extensively explored since the discovery of three independently inherited R genes, namely, *Pia*, *Pii*, and *Pik*, during the 1960s. R genes are distributed across 11 chromosomes of the rice genome, with a notable clustering on chromosomes 6, 11, and 12, representing 18%, 25%, and 21%, respectively (Ashkani et al., 2016). Since the cloning of the first R gene, *Pib*, 41 R genes have been successfully identified (**Table 2**). Except for *pi21*, which acts as a recessive R gene, the remaining 37 R genes exhibit dominance. All of the genes, excluding *pi21*, *Pi35*, *Pi63*, *Pb1*, and *Pid3-I1*, confer complete resistance. *Pi-d2* encodes a B-lectin kinase domain protein (Chen et al., 2006), while *pi21* encodes a proline-rich protein with a heavy metal domain (Fukuoka et al., 2012), and *Ptr* encodes an atypical protein with an armadillo repeat (Zhao et al., 2018); the remaining genes encode nucleotide-binding site leucine-rich repeat (NBS-LRR) domain proteins. Several R genes, including *Pik*, *Pikm*, *Pik-p*, *Pi1*, *Pike*, *Pi5(t)*, *Pia*, and *Pi-*CO39, contain two NBS-LRR protein structural genes for blast resistance (Cesari et al., 2013). The *Pi5-1*, *Pb1*, *pi21*, and *Pi63* genes are induced by pathogen infection, whereas the remaining genes are constitutively expressed. Most cloned R genes confer resistance against leaf blast at the seedling stage, while only a few, such as *Pb1*, *Pi25*, and *Pi64*, provide resistance to panicle blast (Cao et al., 2019).

**Table 2:** Detailed information about the cloned blast resistance genes in rice.

Encoding protein	Resistance against blast disease	Cloning approach for R gene isolates	Presence of avirulence genes	Cloned gene of blast	Chromosome number	Sources of donor variety	References
Nucleotide-binding site leucine-rich repeat (NBS-LRR)	Complete	Map-based cloning	*AVR-Pi9(t)*	*Pi9(t)*	6	75-1-127	Qu et al., 2006
			*Avr-Piz-t*	*Piz-t*	6	Toride 1	Zhou et al., 2006
			*AVR Pik-m*	*Pik-m*	11	Tsuyuake	Ashikawa et al., 2008
			*Avr-Pib*	*Pib*	2	Tohoku IL9	Wang et al., 1999
			Unknown	*Pi64*	1	Yangmaogu	Ma et al., 2015
			Unknown	*Pik-h*	11	K3	Zhai et al., 2014
			Unknown	*Pik-e*	11	Xiangzao 143	Chen et al., 2015
			Unknown	*Pi1*	11	C101LAC	Hua et al., 2012
			Unknown	*Pi2*	6	C101A51	Zhou et al., 2006
			Unknown	*Pi56*	9	Sanhuangzha	Zhai et al., 2019
			Unknown	*Pig-m*	6	Gumei 4	Yang et al., 2020
			Unknown	*Pi50*	6	Er-Ba-zhan (EBZ)	Su et al., 2015
			Unknown	*Pi37*	1	St. No. 1	Lin et al., 2007
		MutMap-Gap	*AVR-Pii*	*Pii*	9	Hitomebore	Takagi et al., 2013
		Mutant screening	Unknown	*Pish*	1	Shin-2	Takahashi et al., 2010
		–	Unknown	*PiPR1*	4	Unknown	Liu et al., 2020
		–	Unknown	*Pizh*	6	Unknown	Zhai et al., 2019
			Unknown	*Pid3-A4*	6	*Oryza rufipogon*	Lv et al., 2013
	Partial	–	Unknown	*Pi35*	1	Hokkai 188	Fukuoka et al., 2014
		–	Unknown	*Pi63*	4	Kahei	Xu et al., 2014
NBS-LRR protein with NB- ARC domain and LRR domain	Partial	Genomewide association mapping	Unknown	*Pb*2	11	Jiangnanwan	Yu et al., 2022
Coiled-coil–nucleotide binding site leucine-rich repeat (CC- NBS-LRR)	Complete	Map-based cloning	*AVR-CO39*	*Pi-CO39*	11	CO39	Cesari et al., 2013
			*AVR-Pikp*	*Pik-p*	11	K60	Yuan et al., 2011
			*AVR-Pi54*	*Pi54*	11	Tetep/*O. officinalis*	Sharma et al., 2010
			*AVR-Pita*	*Pita*	12	Yashiro-mochi	Bryan et al., 2000
			*Avr-Pi54*	*Pi54rh*	11	*O. rhizomatis*	Das et al., 2012
			*AVR-Pik*	*Pik*	11	Kusabue	Zhai et al., 2011
			Unknown	*Pit*	1	K59	Hayashi and Yoshida, 2009
			Unknown	*Pi5*	9	Moroberekan	Lee et al., 2009
			Unknown	*Pb1*	11	Modan	Hayashi N et al., 2010
			Unknown	*Pi36*	8	Q61Pi5	Liu et al., 2007
			Unknown	*Pi25*	6	Gumei 2	Chen et al., 2011
		MB and mutant screening	*AVR-Pia*	*Pia*	11	Aichi Asahi	Okuyama et al., 2011
		*In silico* analysis	Unknown	*Pi-d3*	6	Digu	Shang et al., 2009
	Partial	–	Unknown	*Pid3-11*	6	MC276	Inukai et al., 2019
Proline-rich metal-binding protein	Partial	–	Unknown	*pi21*	4	Owarihatamochi	Fukuoka et al., 2009
B-lectin receptor kinase	Complete	Map-based cloning	Unknown	*Pi-d2*	6	Digu	Chen et al., 2006
A typical protein with an armadillo repeat	Complete	Map-based cloning	Unknown	*Ptr*	12	Katy	Zhao et al., 2018
LRR-RLK	Complete	–	Unknown	Pi65	12	GangYu129	Wang et al., 2022b

## Donors for resistance against rice blast

The development of resistant rice varieties through genetic improvement is a sustainable option for managing plant diseases. Since there are no genotypes with absolute resistance, the identification of reliable resistance sources must be confined to moderate to high levels of tolerance in the germplasm. There are several such genotypes reported (**Table 3**) that are being used in breeding blast-resistant cultivars. Among the cultivated species, the indica cultivars are reported to show better resistance than the Japonica type (Takahashi et al., 2010; Patroti et al., 2019). Additionally, some accessions of wild species, such as O. *rufipogon*, O. *minuta* and O. *glumaepatula* have been reported to be resistant to blast disease.

**Table 3:** Sources of resistance were identified in genotypes of rice against blast disease.

Genes	Source of resistance	References
*Pi1*	IRBL1-CL; C101LAC (LAC23)	Tsunematsu et al., 2000; Mackill and Bonman (1992)
*Pi2*	C101A51; Fukunishiki	Zhou et al., 2006; Kumar et al., 2019
*Pi3*	Paikantao; IRBL3-CP4; C104PKT	Fukuta et al., 2022; Tsunematsu et al., 2000
*Pi5*(t)	RIL125; RIL249; RIL260 (Moroberekan); IRBL5-M	Lee et al., 2009; Tsunematsu et al., 2000; Wang et al., 1994
*Pi7* (t)	IRBL 7-M; RIL 29 (Moroberekan)	Tsunematsu et al., 2000; Telebanco-Yanoria et al., 2010; Wang et al., 1994
*Pi9(t)*	IR71033–121-15; *Oryza minuta* (wild rice)	Amante Bordeos et al., 1992; Qu et al., 2006; Soujanya et al., 2023
*Pi10*	Tongil	Wu et al., 2005
*Pi11* (t)	IRBL11-Zh; Zhaiyeqing 8	Tsunematsu et al., 2000
*Pi12* (t)	IRBL12-M; RIL 10	Tsunematsu et al., 2000
*Pi19* (t)	IRBL19-A; Aichi Asahi	Tsunematsu et al., 2000
*Pi20*	IRBL20-IR24; ARL 20	Tsunematsu et al., 2000
*Pi21*	Owarihatamochi	Angeles-shim et al., 2020
*Pi25*	Gumei 2	Zhuang et al., 2001; Chen et al., 2011
*Pi26*	Gumei 2	Wu et al., 2005
*Pi27* (t)	Q 14	Zhu et al., 2004
*Pi33*	IR 64, Jaya	Berruyer et al., 2003
*Pi34*	Chubu 32 (Sensho); Chugoku 40 (Japanese upland rice ‘Rikuto-kanto 72’)	Saito et al., 2022
*Pi35*	Hokkai 188 (Reishiko)	Saito et al., 2022
*Pi36*	Q 61	Jiang et al., 2019; Liu et al., 2007
*Pi37*	St. No.1; Q1333	Lin et al., 2007; Jiang et al., 2019
*Pi54*	Tetep, NLR145, improved samba mashuri, isogenic line of MTU1010	Kumar et al., 2018; Swathi et al., 2019; Jamaloddin et al., 2020; Dileep Kumar et al., 2023
*Pi61*(t)	93-11	Ma et al., 2014
*Pi63*	Kahei	Xu et al., 2014
*Pi64*	Yangmaogu	Ma et al., 2015
*Pi68(t)*	INGR15002, derived from the cross PR114/*O. glumaepatula* (IRGC 104387)//2*PR11	Devi et al., 2020
*Pia*	Aichi asahi; Zenith; C104PKT; C101LAC; C101A51; CO39; IRBLa-C; IRBLa-A	Goto et al., 1981; Zeng et al., 2011; Tsunematsu et al., 2000
*Pib*	BL1; Tokohu, IL 9, Koshiihikari; IRBLb-B	Kiyosawa, 1984; Telebanco-Yanoria et al., 2010; Hayashi et al., 2006; Tsunematsu et al., 2000
*Pib^a^*	IRAT 13	Telebanco-Yanoria et al., 2011
*PiCO39* (t)	CO39	Chauhan et al., 2002
*Pid2*	Digu	Chen et al., 2006
*Pid3*	Digu	Jiang et al., 2019
*Pik*	Kusabue; Kanto 51; IRBLk-ka	Zhai et al., 2011; Fukuta et al., 2022; Tsunematsu et al., 2000; Yamada et al., 1976
Pik*^a, b^	F-14-3; F-21-6; F-25-3; F-40-3; F-66-1; KU86	Telebanco-Yanoria et al., 2011
Pik*^c^	NP125; F-14-3^d^; F-25-3^d^; F-66-1^d^	Ling (2001); Telebanco-Yanoria et al., 2010
*Pik-h*	IRBLkh-K3; K3	Kiyosawa (1984); Telebanco-Yanoria et al. (2010); Tsunematsu et al., 2000
*Pik-m*	Tsuyuake, IRBLkp-K60; IRBLkm-Ts	Ashikawa et al., 2008; Tsunematsu et al., 2000
*Pik-p*	HR 22; IRBL kp-k60; K60	Yuan et al., 2011; Tsunematsu et al., 2000
*Pik-s*	IRBLks-S; Fujisaka 5; Shin 2; IRBLks-F5; Zhaiyeqing 8; B40; Caloro	Tsunematsu et al., 2000; Kiyosawa (1984); Telebanco-Yanoria et al., 2010
*Pii*	Fujisaka 5; IRBLi-F5	Tsunematsu et al., 2000
*Pish*	Shin 2; Kusabue; Fuknishiki; IRBLsh-B; Toride 1; IRBLsh-S; BL1	Takahashi et al., 2010; Kiyosawa (1984); Telebanco-Yanoria et al. (2011); Tsunematsu et al., 2000
*Pit*	Tjahaja; K59	Hayashi N et al., 2010; Jiang et al., 2019
*Pita*	Fuhui 2663; K1; C105TTP2L9; Zhaiyeqing 8; Yashiromochi, Taducan; IRBLta-K1; IRBLta-CT2; IRBLta-CP1	He N et al., 2022; Kiyosawa, 1984; Telebanco-Yanoria et al., 2010, 2011; Bryan et al., 2000; Tsunematsu et al., 2000
*Pita^a^*	Metica 1	Telebanco-Yanoria et al., 2011
*Pita - 2*	IRBLta2-Pi; IRBLta2-Re; Reiho; Pi No.4; Shimokita	Hayashi et al., 2006; Telebanco-Yanoria et al., 2011; Tsunematsu et al., 2000
*Pita-2^a^*	IR64	Telebanco-Yanoria et al., 2011
*Piz*	IRBLz-Fu; Fukinishiki; IRBLz-Fu	Tsunematsu et al., 2000
*Piz-t*	Toride 1; IRBLzt-T	Zhou et al., 2006; Tsunematsu et al., 2000
*Piz-5*	C101A1 (5173); IRBLz5-CA	Mackill and Bonman, 1992; Telebanco-Yanoria et al., 2011; Tsunematsu et al., 2000
*Pb1*	Asanohikari; Modan	Fujii et al., 1999; Hayashi K et al., 2010; Saito et al., 2022
*Ptr*	Katy	Bryan et al., 2000

### Breeding approaches

Rice breeders employ various strategies to combat blast disease, focusing on the development of resistant cultivars through both conventional and molecular methods. These approaches are designed to improve the longevity of resistance and respond to the changing virulence of *Magnaporthe oryzae*. The following essential breeding techniques are discussed below.

### Traditional breeding

Conventional breeding is one of the oldest methods vital for developing new genetic variants, including blast-resistant rice varieties. This approach depends on interactions between genotype and the environment and helps in phenotypic selection among cultivars. In this scenario, breeders considered factors such as the pathogen’s race, the plant genotype, and resistance (qualitative or quantitative) to disease (Nizolli et al., 2021). Conventional methods are crucial for ensuring genetic diversity, conserving wild germplasm, hybridization, and induce mutations. Over the past 30 years, traditional breeding has produced elite cultivars of IRRIs with a wide array of disease-resistance genes. Various techniques, such as the pedigree method, backcrossing, recurrent selection, and mutation breeding have been frequently employed in conventional breeding programs in recent decades (**Table 4**).

**Table 4:** Successful examples of rice varieties developed through conventional breeding methods against blast disease.

Targeted trait	Genes	Applications	References
Blast resistance	*Pi54*	IR72 variety developed by backcross method	Meher et al., 2023
	*pi21*	Sensho variety was introgressed into an indica breeding line IR63307-4B- 13-2 through backcross breeding method.	Angeles shim et al., 2020
	*Pi54*	The International Rice Research Institute (IRRI) developed a resistant variety against blast through backcross method in IR64 rice variety.	Reinke et al., 2018
	*Pi54*	Indian Council of Agricultural Research (ICAR) developed Swarna Sub1 variety in rice through pedigree method	Ellur et al., 2016
	*Pi1, pi2, Pi9(t), pi20, pi21*	Teqing China variety of rice	Wu Y et al., 2015
	*Pi1*, *Pi2*	A new cytoplasmic male sterile line, Rongfeng 3A, with *Pi1*, *Pi2* was successfully developed through successive backcross breeding	Fu et al., 2012
Resistant to neck blast and susceptible to leaf blast		Norin 6, Norin 22, and Norin 23 were developed by hybridization	Haque et al., 2021

The pedigree method is well-suited for determining traits controlled by major genes. It is extensively used in rice improvement to develop resistance to insects and diseases (Srivastava et al., 2017). However, a major drawback is the time-consuming process of evaluating lines throughout the growing season and maintaining records for selection at maturity. This method requires an interaction between genotype and environment on trait expression. This method may not be the most efficient for traits governed by multiple genes. Backcrossing is a widely employed technique in rice breeding for the introduction or substitution of a target gene from a donor parent to a recipient. This method aims to reduce the donor genome content in progeny, offers a precise way to enhance varieties across multiple traits. Backcross breeding has been widely adopted in southern and southeast Asia as a breeding strategy to improve the resistance of elite varieties such as KDML105, Basmati, and Manawthukha to blast. In addition to backcross breeding, recurrent selection is another method in rice breeding for disease control. This approach facilitates shorter breeding cycles and more precise genetic gains, while promoting a broad range of genetic diversity in breeding lines. Several blast-resistant cultivars, such as the upland cultivar CG-91, have been developed through recurrent selection. Numerous major genes, including *Pib*, *Pita*, *Pia*, *Pi1*, *Pikh*, *Pi2(t)*, and *Pi4(t)*, have been successfully identified and introgressed into rice varieties for blast resistance *via* conventional breeding methods (Miah et al., 2017). Mutation breeding in rice complements conventional breeding by effectively improving major traits such as agronomic characteristics, resistance to pests and diseases, and grain quality parameters. This method is particularly valuable for generating new alleles to develop new varieties. The incorporation of a blast resistance gene into the high-yielding variety Ratna (IR8/TKm 6) was achieved through chemo-mutagenesis (Kaur et al., 1976) using 0.1% and 0.2% ethyl-methane sulfonate (EMS). In China, the mutant rice variety Zhefu, characterized by high resistance to rice blast, was developed through gamma-ray irradiation of the variety Simei 2 (Ahloowalia et al., 2004). Major blast resistance genes including *Pib*, *Pita*, *Pia*, *Pi1*, *Pikh*, *Pi2*, and *Pi4* have been successfully introduced into rice varieties *via* conventional breeding programs. The durability of multiline varieties is influenced by the rate at which blast races develop, the proportion of lines present in a mixture and the size of the planted area. Attempts have been made to develop multiline varieties using blast resistant isogenic lines for “Nipponbare” (Higashi et al., 1981; Horisue et al., 1984), “Toyonishiki” (Nakajima, 1994) and “Sasanishiki” (Matsunaga, 1996). Studies have confirmed that blast control is achieved through the use of multiple line varieties (Nakajima et al., 1996). Specifically, the “Sasanishiki” multiple-line variety has been commercially grown on a market scale since 1995. In addition, new isogenic lines have been developed and a detailed examination of the races of the blast pathogen has been carried out, which is crucial for stable use (Ashizawa et al., 2007). A cross combination of Koshihikari blast-resistant isogenic lines (BLs) was developed (Ishizaki et al., 2005). The BLs were developed by crossing with Sasanishiki (*Pia*), Todorokiwase (*Pii*), *Pi4* (*Pita*-2), Niigatawase (*Piz*), Koshiminori (*Pik*), Tsuyuake (*Pik-m*), Toride 1 (*Piz-t*) and BL1 (*Pib*) as the donor parent respectively, and then repeated back-crossings with “Koshihikari” as the pollen parent were performed.

Gene strategy implementation involves utilizing different blast resistance mechanisms in various rice varieties and arranging them in specific temporal or spatial patterns. Rice cultivation practices incorporate seasonal and regional preferences for location-specific varieties. This approach allows for the development of distinct varieties using diverse blast resistance sources. Even within varieties used for a particular season, those with different maturity periods should incorporate unique blast resistance sources. This method slows the evolution of new virulent races and enhances the durability of blast resistance in current varieties. Among various strategies, deploying distinct genes in different maturity groups may improve the longevity of blast resistance in newly developed rice varieties. However, traditional resistance breeding has notable limitations, including extended breeding cycles, inefficient selection processes, and challenges in distant crossing. These drawbacks result in a lag between the creation of new resistant cultivars and the emergence of virulent pathotypes of the causative pathogen.

## Molecular approaches

### QTL mapping and GWAS

**Figure 4:**
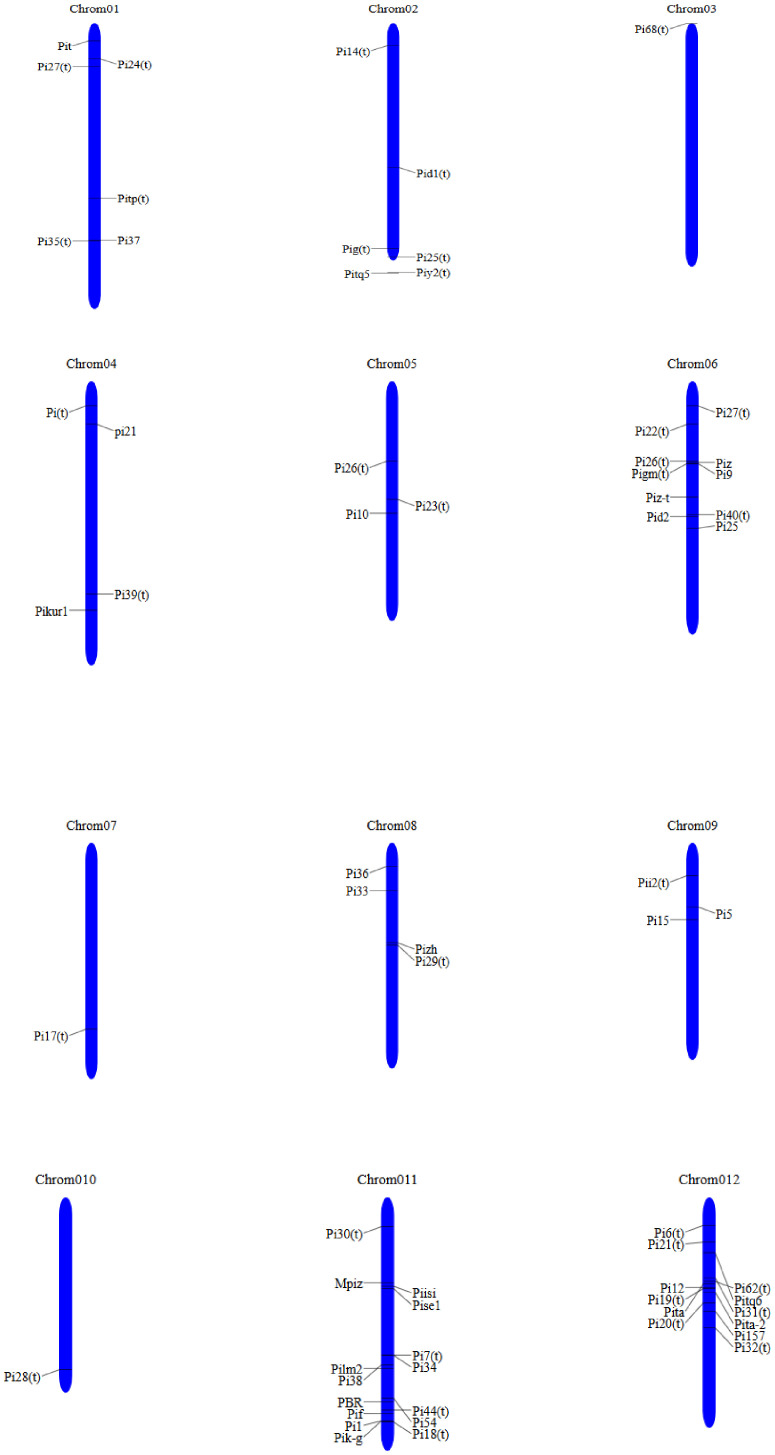
Distribution of QTLs mapped within rice genome against blast disease in rice.

Rice germplasm harbors both qualitative and quantitative types of blast resistance genes. Blast resistance can be categorized into complete resistance, governed by major genes (R genes) and exhibiting race specificity; and partial resistance, governed by numerous genes known as quantitative trait loci (QTLs). Partial disease resistance offers durable protection against a wide range of pathogens, promising avenue for sustainable rice production in future. QTL detection serves as a valuable tool for mapping major or minor genes responsible for disease resistance (Mora et al., 2016). The mapping and tagging of QTLs linked to blast resistance can facilitate the cloning of major disease resistance genes and aid in marker-assisted breeding programs for the development of resistant cultivars. Many major blast resistance genes are qualitative in nature and have been identified and mapped within the rice genome (**Figure 4**) (Ashkani et al., 2016).

Genome-Wide Association Studies (GWAS) are a powerful tool used to identify genetic loci associated with blast resistance in rice by analyzing genetic variations across diverse rice populations. This approach has revolutionized resistance breeding by uncovering novel resistance (R) genes and quantitative trait loci (QTLs) associated with blast resistance. GWAS evaluates the statistical association between genetic markers (e.g., SNPs) and phenotypic traits (e.g., resistance to blast disease). QTLs are identified through GWAS and can be targeted for breeding. The discovery of new loci in wild rice species, such as *Oryza rufipogon* and *Oryza nivara*, offers novel sources of resistance. In the United States, a set of 151 accessions were used with 156 SSR markers for association with seed weight, plant height, and heading against blast resistance (*R*) genes, *Pi-ta* marker in rice (Wang et al., 2015). GWAS along with RNA sequencing analysis was performed to identify novel marker–trait associations against blast resistance in rice, where *Pi5* and *Pi56(t)* was on chromosome 9 and 127 associations and 283 upregulated genes were revealed. The expression level increased after fungal inoculations; 401 downregulated genes significantly decreased against blast disease (Lu et al., 2019). In *Japonica* rice varieties from European countries, 311 accessions were screened and 14 marker–trait associations for blast resistance were identified using both field and growth chamber screenings (Volante et al., 2020). A nitrogen-induced susceptibility (NIS1) locus was analyzed in 139 Japonica rice strains by using the GWAS technique and conferred blast resistance by the identification of novel loci (*NIS2*, *NIS3*, and *RRobN1* on chromosomes 5, 10, and 6) to be involved in the rice against blast fungus under different nitrogen regimes (Frontini et al., 2021). The significant associations were identified as the candidate loci in 48 accessions of rice for the blast resistance in rice that will serve as an important genetic resistance source to be introduced into an elite rice line in future breeding programs for deciphering blast resistance in rice. This GWAS helped to uncover significant gene regions which encode proteins to resist blast infection in rice plants (Barua et al., 2024). GWAS in rice identified 43 QTLs significantly associated with resistance to panicle blast genes for *OsAKT1*, *OsRACK1A*, *Bsr-k1*, and *Pi25*/*Pid3* (Jinlong et al., 2024). Pangenome-wide association study (panGWAS) was carried out on nine blast resistance-related phenotypes using 414 international diverse rice accessions from an international rice panel, and 74 QTLs associated with rice blast resistance were identified. The significant potential QTL (*qPBR1*–6 candidate genes) confers resistance to both panicle and leaf blast throughout their growth period, and 3,311 differentially expressed genes are involved against blast resistance (Wang J et al., 2024). From these references, it can be inferred that GWAS loci are combined with known *Pi-genes* to develop varieties with durable, broad-spectrum blast resistance in rice.

### Marker-assisted selection

Marker-assisted selection (MAS) is a tool and often controlled by a single or few genes. The MAS leverages the specific interaction between resistance (R) genes and avirulence (*AVR*) genes in host–pathogen interactions, thereby enhancing blast control (Petit-Houdenot and Fudal, 2017). By identifying molecular markers linked to desired traits, MAS improves the efficiency of conventional breeding methods—for instance, a set of SSR markers (RM168, RM8225, RM1233, RM6836, RM5961, and RM413) associated with blast resistance has been identified and could be utilized in MAS programs. These molecular markers, combined with MAS strategies, play a critical role in the development of durable blast-resistant plant varieties. The three rice varieties, namely, BRRI dhan48, BRRI dhan58 (recurrent parent), and IRBL9-W (Donor parent), were crossed for blast resistance, and introgression of genes was confirmed by marker-assisted selection (Nadim et al., 2024). The drought variety of rice Huhan 1516 had the blast *Pi*2 gene introgressed by using this technique, where Huhan 1509 was the donor parent (Li A et al., 2024). The water-saving and drought-resistant rice core parents were Hanhui 3, BL675-1-127, and B5. BL5 carries the *Pi1* and *Pi2* genes, BL675-1-127 carries the *Pi9(t)* gene, and *B5* carries the *Bph14* and *Bph15* genes; these were introgressed through MAS (Liu G et al., 2024). Fengshun et al. (2024) had genes introduced into the *Japonica* rice cultivar Anhui for blast resistance genes *Pita*, *Pi5(t)*, *Pi1*, *Pia*, *Pik*, *Pi54*, and *Pb1.* To introduce the broad-spectrum blast resistance gene *R*6 into the early *indica* rice thermosensitive genic male sterile (TGMS) line HD9802S was used by employing MAS (Chen et al., 2023). The Mushk budji cultivar was used as recipient parent where the blast resistance genes *Pi9(t)* and *Pi54* were introgressed (Shafi et al., 2023). A set of 119 rice main varieties (94 *Japonica* and 25 *indica*) were used to introduce the 14 major blast resistance genes *Pit*, *Pish*, *Pib*, *Pi1*, *Pia*, *Pi54*, *Pita*, *Pi9(t)*, *Pi2*, *Pikm*, *Pigm*, *Pi5*, *Pb1*, and *Piz-t* through marker-assisted selection, which were screened by SSR markers (Qi et al., 2023). The *Pi9(t)*, *Pi5*, and *Pi54* genes were introduced into Huhan 1s and Huhan 74S (Liu et al., 2021). According to Wu et al. (2016), the Yangdao 6 rice variety was where broad-spectrum durable resistance genes *Piz-t*, *Pi2*, *Pigm*, *Pi40*, *Pi9(t)*, and *Piz* were introgressed and validated with SSR markers. From these inferences, we can conclude that blast resistance genes like *Pib*, *Pi1*, *Pia*, *Pi54*, *Pita*, *Pi40*, *Pi9(t)*, *Pi2*, *Pikm*, *Pigm*, *Pi5(t)*, *Pb1*, and *Piz-t*, etc., were introgressed into different suitable recipient cultivars, and these resistance varieties can further help in the development of new varieties that are useful in crop breeding programs.

### Marker-assisted backcross breeding

Marker-assisted backcrossing (MABB) is a simplified breeding method in which molecular markers are used to precisely target specific genetic loci, which reduces the length of donor segments containing these loci and allows us to efficiently recover desired traits from the recurrent parent genome. The aim of this approach is to transfer targeted genes while reducing donor segment size and retaining recurrent parental characteristics (Hasan et al., 2015). MABB offers precision, efficiency, and time savings over traditional backcrossing methods by employing tightly linked molecular markers for key traits. This approach has been widely adopted to transfer resistance genes into popular rice varieties worldwide (**Table 5**). Numerous studies have demonstrated the efficacy of marker-assisted selection in developing new rice varieties. The blast and bacterial blight resistant lines, two blast resistance genes (*Pi9(t)* and *Pb1*) and three bacterial blight resistance genes (*xa5*, *xa13*, and *Xa21*), were pyramided in the background of premium quality rice variety BRRI dhan63 through marker-assisted backcross breeding. *Pi9(t)*-US2 and Pb1-US2 were used as donor parents for *Pi9(t)* and *Pb1*, respectively, and for *xa5*, *xa13*, and *Xa21*, IRBB60 was used as the donor parent (Nihad et al., 2024). Sowmiya et al. (2024) examined that the resistant varieties through MABB were blast resistance gene *Pi54* introgressed into ADT43 from RP-Bio-Patho-2. The mega rice variety with high-yielding Swarna lines was introgressed with blast and blight genes *Pi54* and *Xa21* into the near-isogenic line of improved samba mature through this MABB technique (Kousik et al., 2024). Dileep Kumar et al. (2023) reported on the introgression of blast resistance gene *Pi54* (isogenic line of MTU 1010 as donor parents) into Jaya rice variety as the recurrent parent.

**Table 5:** Successful examples for introgression of blast-resistant genes in rice through marker- assisted selection (MAS) and marker-assisted backcross breeding (MABB).

Approach	Name of marker linked to trait	Blast resistance genes	Developed lines/alter the function of blast resistance (introgression into)	References
MABB	Indel	*Pi39*	Chinese cultivar Q15	Hua et al., 2015
	SSR	*Pi1*, *Pi2*	Intan variety and BPT5204	Hegde and Prashanthi, 2016
		*Pi46*, *Pita*	Hang hui 179 (HH179)	Xiao et al., 2016
		*Pi2* and *Pi5(t)*	*Pi2* from C101A51 and *Pi5* from IRBL-5 M, into BPT-5204 (Samba Mahsuri)	Sagar et al., 2017
		*Pi54*	Samba Mahsuri	Kumar et al., 2018
		*Pi2*, *Pi46*, and *Pit a*	H4 and R175	Xiao et al., 2019
		*Piz*, *Pi2*, and *Pi9(t)*	Malaysian rice variety Putra-1	Chukwu et al., 2019
		*Pi1*, *Pi2*, and *Pi54*	Swarna-Sub 1 lines	Patroti et al., 2019
		*Pi54*	JGL1798 (Jagtial Sannalu)	Swathi et al., 2019
		*Pi54*	ASD 16 and ADT 43	Ramalingam et al., 2020
		*Pi54*, *Pi1*	TH and NLR145 rice varieties	Jamaloddin et al., 2020
		*Pi9(t)*	Himalaya 741 cultivar	Rathour et al., 2022
		*Pi9(t)* and *Pi54*	Ranbir basmati	Pote et al., 2022
		*Pi9(t)* and *Pi54*	Mushk budji cultivar	Shafi et al., 2023
	SNP	*Pi9(t)*, *Pizt*, *Pi54*	*Pi9(t)*, *Pizt*, and *Pi54* blast resistance genes into japonica rice 07GY31	Xiao et al., 2016
		*Pi54, Pi1*, and *Pita*	*Mushk Budji*	Khan et al., 2018
		*Pi9(t)*, *Pi1*, and *Pi2*	HZ02455 and HZ02411 rice cultivars	Dasari et al., 2022
		*Pib*, *Pita*, *Pik*, *Pi9(t)*, *Pi1*	*Japonica* Italian rice variety	He Z et al., 2022
		*Pi54*	TN 1 and Jaya	Dileep Kumar et al., 2023
MAS	SSR	*Pita*, *Pi5(t)*, *Pi1*, *Pia, Pik, Pi54, Pb1*	*Japonica* rice cultivar Anhui	Fengshun et al., 2024
		*Pi54* and *Pi9(t)*	RP5933-1-19-2R	Soujanya et al., 2023
		*Pig-m*	geng/japonica rice	Feng et al., 2022
		*Pi2*	DRR 9B and Samba Mahsuri	Singh et al., 2023
		*Pi9(t)* and *Pi54*	CO 51	Samuthirapandi et al., 2023 and Thulasinathan et al., 2023
		*Pi9(t)*, *Pi5*, *Pi54*	Huhan 1S and Huhan 74S	Liu et al., 2021
		*Piz-t*, *Pi2*, *Pigm*, *Pi40*, *Pi9(t)*, *Piz*	Yangdao 6	Wu et al., 2016
		*Pi1*, *Pi2*, *Pi33*	Russian rice varieties	Usatov et al., 2016

The sd1 gene for semi-dwarfism and the *Pi9(t)* and *Pi54* genes for blast resistance were incorporated into a traditional basmati rice variety, Ranbir Basmati (Pote et al., 2022). Dasari et al. (2022) revealed that blast resistance was developed by introducing the *Pi9(t)*, *Pi1*, and *Pi2* genes in HZ02455 and HZ02411 rice cultivars, as confirmed using SNP markers. The Japonica rice cultivar was also introgressed with blast resistance genes *Pib*, *Pita*, *Pik*, *Pi9(t)*, and *Pi1*, as validated by SNP markers (He Z et al., 2022). The broad-spectrum resistance locus *Pi9(t)* was transferred from a Basmati donor, PB1637, into the cold-tolerant variety Himalayan 741 (Rathour et al., 2022). The *Pi1* and *Pikh* blast resistance genes were incorporated into the Jyothi and Kanchana rice varieties from Parambuvattan through marker-assisted backcross breeding (MABB) (Anusha, 2022). Tellahamsa, a cold-tolerant variety, served as the recurrent parent, while Improved Samba Mahsuri (*Xa21* and *xa13*) and NLR 145 (*Pi54* and *Pi1*) were selected as donor parents for introgression using MABB (Jamaloddin et al., 2020). In ASD 16 and ADT43, the *Pi54* blast genes were introduced and confirmed by SSR markers (Ramalingam et al., 2020). Sagar et al. (2020) revealed that the introgression of two genes, each governing resistance to major rice diseases, bacterial blight (BB) (*xa13* and *Xa21*) and blast (*Pi2* and *Pi54*), was achieved in the popular basmati cultivar Pusa Basmati 1509 through marker-assisted backcross breeding (MABB). The Malaysian rice variety Putra-1 harbors blast resistance genes *Piz*, *Pi2*, and *Pi9(t)* (Chukwu et al., 2019). Xiao et al. (2016) reported the introgression of the *Pi9(t)*, *Pizt*, and *Pi54* blast resistance genes into japonica rice 07GY31. Hence, these selected plants can be forwarded for further generations to develop high-yielding blast-resistant rice lines. Hence, addressing blast resistance in genes is typically focused on the successful integration of resistant genes *Piz*, *Pi2, Pi54*, *Pi1*, and *Pi9(t)* into high-yielding or any locally adapted cultivars through MABB which is helpful to enhance the ability to withstand the pathogen *M. oryzae*. Consequently, this strategy serves as an effective tool in modern rice breeding programs, significantly enhancing blast resistance while ensuring high productivity and climate adaptability.

### Omics approaches

Generally, omics approaches, including genomics, transcriptomics, proteomics, and metabolomics, have drastically revolutionized the study of blast resistance in rice by providing comprehensive insights into the genetic and molecular mechanisms underlying host–pathogen interactions (Greenwood et al., 2024) (**Table 6**). Genomic studies have identified key genes and genomic regions associated with blast resistance in rice. Genome-wide association studies (GWASs), quantitative trait loci (QTLs) mapping, and comparative genomics are often used to identify candidate genes and alleles conferring resistance (Wei et al., 2021). High-throughput genotyping techniques such as SNP arrays and next-generation sequencing facilitate the identification of genetic markers associated with resistance traits. Genome-wide association studies (GWASs) have been utilized to identify natural allelic variations associated with blast resistance across diverse rice germplasms, offering valuable insights into the genetic framework governing resistance (Abhijith et al., 2022). Genomic selection (GS) leverages genome-wide marker information to predict the breeding value for blast resistance in rice breeding programs. It enables the early selection of superior genotypes based on genomic estimated breeding values (GEBVs) for crop improvement (Tabassum et al., 2021). Transcriptomic studies have provided dynamic gene expression changes against rice blast infection. RNA sequencing (RNA-seq) and microarray analysis have been used to profile gene expression patterns during different stages of infection and in response to various pathogens (Zhu et al., 2024) and are also helpful in identifying differentially expressed genes involved in defense signaling, pathogen recognition, and secondary metabolite biosynthesis, providing insights into the molecular basis of rice defense mechanisms against blast disease (Chandrakanth et al., 2024). Transcriptomics facilitates identifying and characterizing transcription factors (TFs) and regulatory elements involved in blast resistance. TFs such as WRKY, NAC, and bZIP families are key regulators of defense gene expression and play crucial roles in coordinating immune responses (Thapa et al., 2024).

**Table 6:** Various achievements of omics approach against blast resistance in rice.

Omics approaches	Achievement	References
Genomics	o Identification and characterization of blast resistance genes from diverse rice germplasm o Discovery of novel blast resistance genes and QTLs using GWAS and transcriptomic analyses o Adoption of MAS for introgression blast resistance genes into elite cultivars o Identification of blast resistance genes and QTLs from wild rice relatives and traditional landraces using genomic tools	Greenwood et al., 2024; Liang et al., 2022; Sheoran et al., 2021
Transcriptomics	o Development and release of blast-resistant rice varieties such as IR64-Sub1 and Swarna-Sub1 o Development of blast-resistant rice varieties through gene pyramiding and genomic selection o Identification and characterization of novel blast resistance genes are being pursued through rigorous transcriptomic and proteomic analyses	Hafeez et al., 2024; Yang et al., 2024; Xu et al., 2022; Patel et al., 2023
Proteomics	o Identification of pathogen effectors and host targets associated with rice blast resistance using proteomics o Characterization of metabolic changes and defense- related metabolites in blast-resistant rice varieties o Discovery of key regulatory proteins and signaling pathways involved in blast resistance through proteomic analysis	Liu Y et al., 2024; Kumari et al., 2023; Zhou H et al., 2022; Nawaz et al., 2020
Metabolomics	o Identification of key metabolic pathways and regulatory proteins contributing to blast resistance in rice o Employing metabolomics to identify metabolite biomarkers associated with blast resistance in various rice cultivars	Hafeez et al., 2024; Eides et al., 2024; Wu et al., 2021

Proteomic studies are helpful in identifying the complex network of proteins involved in rice defense responses against blast. High-throughput proteomic techniques, including quantitative proteomics and phospho-proteomics, are being used to identify key signaling components and defense proteins (Zhang et al., 2022). Recent proteomic analyses have revealed post-translational modifications and protein–protein interactions that regulate the activation of defense pathways in rice upon blast infection (Wang et al., 2022a) and also the identification of defense-related proteins, such as chitinases, peroxidases, and pathogenesis-related (PR) proteins that are upregulated in response to blast infection. Techniques such as two-dimensional gel electrophoresis (2-DE) and liquid chromatography–mass spectrometry (LC–MS/MS) facilitate the comprehensive profiling of proteins, leading to the discovery of key defense proteins and signaling pathways against rice blast disease (Zhao et al., 2021). Metabolomics is the analysis of small molecules in biological systems, elucidating the metabolic changes underlying blast resistance in rice. Advances in metabolite profiling techniques are enabling the identification of defense-related metabolites and metabolic pathways, phytohormones, and redox-active compounds helpful in disease response against blast in rice (Shi et al., 2024). High-throughput analytical techniques such as liquid chromatography–mass spectrometry (LC–MS) and gas chromatography–mass spectrometry (GC-MS) allow for the comprehensive profiling of metabolites, facilitating the discovery of biomarkers and key metabolic pathways. Metabolomic studies have shown changes in the levels of defense-related metabolites, such as phytoalexins, phenolics, volatile organic compounds (VOCs), and flavonoids, in response to blast infection. Numerous studies have been done by using these multi-omic approaches. In rice, overexpressing phytochrome-interacting factor-like 1 (*OsPIL1*) rice lines were evaluated in terms of their impact on growth, grain development, and resistance to *Magnaporthe oryzae*. Multi-omics analysis (RNA-seq, metabolomics, and CUT&Tag) and RT-qPCR validated the *OsPIL1* target genes and key metabolites (Zhao et al., 2024). Multi-omics approach, especially proteomics, was used to comprehensively analyze *MoKin*1 function, and the results revealed that *MoKin*1 affected the cellular response to endoplasmic reticulum stress (ER stress), of which the downregulated proteins in *ΔMokin1* mutant were enriched mainly in response to ER stress triggered by the unfolded protein. Therefore, the phosphorylation of various proteins regulating the transcription of ER stress-related genes and mRNA translation was significantly downregulated (Zhang L et al., 2024). Integration of transcriptomic, proteomic, and phosphor-proteomic analysis of Mowanggu was performed after inoculation with *M. oryzae*, revealing that differentially expressed genes and proteins were upregulated and significantly enriched in protein phosphorylation, peroxisome, plant–pathogen interactions, phenylpropanoid metabolism, phenylalanine biosynthesis pathways, reactive oxygen species (ROS), glycolysis, MAPK signaling pathways, and amino acid biosynthesis against rice blast resistance (Peng et al., 2023). Hence, from these, it was concluded that omics approaches *viz*., genomics, transcriptomics, proteomics, and metabolomics, have revolutionized research on blast resistance in rice required for GWAS (identification of resistance, susceptibility genes, QTLs) linked to blast, paving the way for marker-assisted breeding and genome editing, differential gene expression, and identification of proteins (pathogenesis-related proteins, ROS pathways), secondary metabolites, and signaling molecules that contribute to the development of resistant lines against blast disease.

### Allele mining

Allele mining is a widely employed molecular technique for identifying allelic variations or novel alleles within a targeted gene. This approach involves thoroughly characterizing a large set of germplasm collections used for allele mining. Tilling and eco-tilling techniques are used to identify induced point mutations in the targeted gene by heteroduplexes of alleles during DNA replication process for identifying allelic variations. Sharma et al. (2012) reported the allelic variation for genes responsible for rice blast resistance, such as *Pi-ta*, *Pi-kh*, and *Pi-z(t)*, among Indian land races using allele mining technique. Their findings revealed a substantial variation in *Pi-kh* and *Pi-z(t)* alleles compared to *Pi-ta* alleles. Effective allele mining uses genetically diverse materials with prior knowledge of gene sequence information. This approach aids in the detection of superior alleles from both wild and cultivated rice species available thus far for blast resistance genes. A large-scale screening of new blast resistance alleles was conducted across 2,000 rice accessions from major rice-producing areas in China. Sequence-based allele mining was used to identify the allelic variants of major rice blast resistance genes at the *Pi5* locus of chromosome 9. Six novel alleles were identified from 64 accessions, and 153 accessions showed moderate resistance against blast (Zhou Y et al., 2024). The alleles from seven varieties showing high resistance were selected for transformation into the susceptible variety J23B to construct near-isogenic lines (NILs). There is a large-scale screen of rice blast resistance in about 2,000 rice accessions; among them, 247 accessions showed at least medium resistance, and seven novel *Pik* alleles were identified as blast resistant. The rate of *Pik-R0/ME/7017* donors was greater than 80% (Ying et al., 2022).

These NILs showed resistance in a field test in Enshi and Yichang, indicating that the seven novel rice-blast-resistance tandem-repeat regions at the *Pi2/Pi9(t)* locus of chromosome 6 could potentially serve as a genetic resource for molecular breeding of resistance to rice blast (Zhou et al., 2020). Allele mining for blast-resistant gene *Pi9(t)* was performed in 338 rice landraces, among them 136 polymorphic sites comprising of transitions, transversions, and insertion and deletions (InDels) were identified in the 2.9-kb sequence of *Pi9(t)* alleles (Imam et al., 2016). The *AC134922* locus is nucleotide-binding-site leucine-rich-repeat (*NBS*-*LRR*) gene family in rice genome where six rice blast resistance (*R*) genes have been cloned from this locus and two resistance candidate genes, *Pi34* and *Pi47*, are also mapped. A total of 22 genes from 12 cultivars based on allele-mining strategy was cloned at this locus, and six rice blast *R* genes were identified, with four of them recognizing more than one isolate (Wang et al., 2014). PCR-based allele mining for blast resistance gene *Pi54* from six cultivated rice lines and eight wild rice species was carried out to understand its structural variation and its impact on the phenotypes in Tetep (*Pi54* genes) in which the sequence analysis showed more interspecies variation of cultivated and wild species. The structural analysis of alleles showed the presence of a variable number of open reading frames (0–2) principally having point mutations in the leucine-rich repeats (LRR) regions, and these resistance alleles can be used in the effective management of rice blast disease through gene pyramiding (Kumari et al., 2013). The identification of novel alleles of rice blast resistance genes *Pikh* and *Pita* genes with linked markers RM206, TRS26, TRS33, YL153, YL154, YL155, and YL87 for *Pita* was also used to screen materials based on marker profiles to downsize the number of genotypes for allele mining (Ramkumar et al., 2010). Therefore, it concluded that allele mining serves as a cornerstone helpful in identifying and characterizing genetic variations in blast resistance genes that contribute to combat the fungal pathogen *M. oryzae*.

### Genomic selection

Genomic selection (GS) is an advanced breeding approach that utilizes genome-wide genetic information to predict the performance of rice cultivars. This technique has revolutionized rice breeding, enabling the rapid development of blast-resistant varieties by predicting and selecting resistance traits without the need for extensive phenotypic evaluation to improve breeding efficiency by predicting the individuals’ genetic potential based on their genome-wide marker data (Meuwissen et al., 2001). The main principle of this technique is using a training population with known phenotypic and genotypic data to develop prediction models. These models are then applied to selection candidates to predict their resistance to blast disease. GS allows the simultaneous selection of multiple quantitative trait loci (QTLs) associated with blast resistance, speeding up the breeding process and enabling the accumulation of favorable alleles (Escola et al., 2023). Genomic selection integrates genomic information from resistance genes, such as *Pi* genes (*Pi9(t)*, *Pi54*, and *Pi33*), which are associated with blast resistance. High-throughput genotyping methods like SNP arrays and next-generation sequencing are used to identify markers linked to blast resistance traits. Models are trained on datasets combining phenotypic resistance data (from artificial or natural blast infections) with genome-wide marker profiles (Younas et al., 2024).

Advanced statistical techniques, such as ridge regression best linear unbiased prediction (RR-BLUP), Bayesian models, and machine learning algorithms, improve the accuracy of predictions (Xu et al., 2021). GS complements traditional marker-assisted selection (MAS) by allowing breeders to select complex traits controlled by multiple genes, such as partial resistance to blast, which is often governed by polygenes (Hickey et al., 2017). The main components of GS are (i) utilizing genome-wide markers simultaneously to develop a genotype–phenotype relationship model in one population (called training population) accounting for genome-wide linkage disequilibrium (LD) among markers and (ii) predicting the genomic estimated breeding values (GEBV) based on the model in future candidates of other related populations (called breeding population) (Heffner et al., 2009). The success of GS depends on the accuracy of prediction and predictability of models adapted to different crops (Crossa et al., 2017). A set of 162 rice lines from USDA and 237 African lines was evaluated for blast resistance as determined by genomic estimated breeding values (GEBVs) by RR-BLUP model and confirms that the accuracy of genomic selection for blast resistance in rice varies from germplasm to germplasm which ranges from 0.29 to 0.59 (Balimponya, 2015). Genomic selection studies by using GBLUP statistical method in 161 African rice accessions found resistance against blast disease in rice (Huang et al., 2019). Hence, it confirms that different statistical models are helpful for a selection of traits controlled by multiple genes, QTLs against blast resistance in rice, which leads to the development of broad-spectrum varieties by genomic selection.

The International Rice Research Institute (IRRI) has implemented genomic selection to pyramid blast resistance genes (*Pi9(t)* and *Pi54*) into high-yielding rice varieties. This approach has successfully developed improved varieties like *NSIC Rc222* with enhanced resistance and better yield potential (www.irri.org). Chinese researchers also used this technique to identify and incorporate QTLs associated with broad-spectrum blast resistance into elite varieties. Hybrid rice breeding programs in India and Southeast Asia use genomic selection to predict blast resistance in parental lines (Xiao et al., 2021). This has enabled the development of hybrids with improved blast resistance and high yields. Wild relatives of rice (*Oryza rufipogon* and *Oryza nivara*) have been incorporated into GS programs to introduce novel blast resistance genes (Balimponya, 2015).

### Speed breeding

Speed breeding is an innovative technique designed to accelerate the traditional breeding cycle, enabling the rapid development of rice varieties with enhanced resistance to diseases like blast (*Magnaporthe oryzae*) (Ahmar et al., 2020). The approach leverages controlled environments, such as growth chambers or greenhouses, to shorten generation time by optimizing environmental factors, which is helpful for the deployment of blast-resistant varieties. Speed breeding aims to increase the rate of genetic improvement by shortening the time, particularly useful for traits like disease resistance, which needs continuous improvement due to evolving pathogens (Sharma et al., 2022). By optimizing photoperiod, light intensity, temperature, and humidity, this technique allows rice plants to flower and mature more quickly than under traditional field conditions. Growth chambers or controlled greenhouses that allow for extended day-length conditions (up to 22 h of light per day) and higher temperatures promote faster plant development (Jahne et al., 2020). Speed breeding can be integrated with MAS to identify plants carrying specific *Pi-genes* or other resistance loci (Chimmili et al., 2022). The University of Queensland has developed a speed breeding platform that allows researchers to rapidly generate rice lines with enhanced resistance to diseases like blast. The International Rice Research Institute (IRRI) and the University of Sydney use speed breeding methods to shorten breeding cycles and rapidly incorporate blast resistance genes into high-yielding varieties. Speed breeding allows breeders to introduce blast resistance genes into elite varieties much faster than conventional methods. The breeding cycle is shortened from 5 to 6 years to as little as 8–12 months, allowing the quicker deployment of resistant varieties to combat blast outbreaks. Faster breeding cycles allow for the pyramiding of multiple resistance genes (*Pi-genes* and others) into a single variety, leading to more durable and broad-spectrum resistance (Zainuddin et al., 2024). Hence, the combination of speed breeding with genomics-assisted breeding (MAS and GWAS) can further accelerate the development of blast-resistant varieties.

### Transgenic approaches

These transgenic approaches are vital in mitigating blast disease and yield loss by enhancing resistance and improving crop resilience. Hence, to address this issue, improving high yield and climate resilience traits can be approached through efficient strategies, i.e., RNA interference, transgenic, and genome editing.

### RNAi interference

RNA interference (RNAi) has emerged as a potent and efficient tool for combating various challenges caused by small microbial organisms such as viruses, bacteria, and fungi. In RNAi, short interfering RNA (siRNA) molecules are used to silence targeted gene expression. In rice blast management, siRNA molecules target essential genes in the fungus, disrupting its growth and virulence (Jain et al., 2017). Tissue-specific gene silencing is induced by employing gene-specific promoters to simultaneously silence several genes. Successful reports have proven the efficiency of RNAi in achieving integrated biotic resistance against major diseases and pests in plants. The dsRNA (PyDCL2–863 bp) was synthesized for the silencing of DCL2 transcript of *P. oryzae* through RNA interference and showed potential for PyDCL2-dsRNA to be developed as a new fungicide for the sustainable disease management of rice blast (Pushpanjie et al., 2024). HIGS (host-induced gene silencing) is used for six genes (CRZ1, PMC1, MAGB, LHS1, CYP51A, and CYP51B) that play important roles in the pathogenicity and development of *M. oryzae*. HIGS vectors were transformed into rice calli through *Agrobacterium*-mediated transformation, and T0, T1, and T2 generations of transgenic rice plants were generated. Following infection with *M. oryzae* of HIGS transgenic plants, the expression levels of target genes were reduced as demonstrated by quantitative RT-PCR. In addition, treating *M. oryzae* with small RNA derived from the target genes inhibited fungal growth. These findings suggest that RNA silencing signals can be transferred from the host to an invasive fungus and that HIGS has the potential to generate resistant rice against *M. oryzae* (Wang and Dean, 2022). A transient dsRNA supplementation system for the targeted knockdown of *MoDES1*, a host-defense suppressor pathogenicity gene from *M. oryzae*, was carried out by spray-induced silencing (Sarkar and Roy-Barman, 2021). Thus, these findings confirm that RNAi-based gene expression approaches are a powerful tool for blast disease control in rice. Spray-induced gene silencing (SIGS) utilizing double-stranded RNA (dsRNA) or small interfering RNA (siRNA) is gaining interest because of its low cost and straightforward preparation in transgenic plants. Once dsRNA is applied to the leaf surface, it can either directly target pathogen cells or be absorbed by plant cells and transferred to the pathogens. This technique is further useful for silencing blast resistance genes and is helpful for the development of broad-spectrum durable resistance varieties in rice.

### Genome editing

Genome editing techniques like CRISPR/Cas9, TALEN, and ZFNs have been used to develop various rice varieties for blast resistance (**Table 7**) (Viana et al., 2019). Among these, CRISPR/Cas9 techniques are more widely used in rice than TALEN and ZFNs. Rapid and adaptable genome modification is a potent approach for gaining comprehensive insights into molecular mechanisms in biological studies. Recently, genome editing facilitated by CRISPR/Cas9 has emerged as a dependable method for genetic manipulation across various biological research fields, including investigations of filamentous fungi. The CRISPR/Cas9 system consists of a Cas9 protein and a single-guide RNA (sgRNA), with the Cas9/sgRNA complex inducing a DNA double-strand break at the intended genomic site. This protocol outlines a fundamental CRISPR/Cas9 methodology, encompassing target sequence design, CRISPR/Cas9 expression vector construction, and transformation for genome editing in *Pyricularia* (*Magnaporthe*) *oryzae*. This approach enables efficient targeted gene disruption, base editing, and reporter gene knock-in without necessitating additional modifications to host components. The protocol also applies to implementing other CRISPR/Cas technologies and diverse functional genomics studies in *P. oryzae*. An 84-bp arginyl (Arg)-tRNA promoter-driven CRISPR/Cas9 system enables efficient and cost-effective gene editing in *P. oryzae*. By using the Mo_tRNA^Arg24^-gRNA-Cas9 cassette, the *Ppg1* gene disruption rate was increased up to 75.9% (Wang R et al., 2024). Developing *Bsr-d1* knockout mutants *via* CRISPR/Cas9 enhances broad-spectrum resistance to rice blast in Northeast China (Zhang Y et al., 2024). CRISPR/Cas9-induced mutations in the *MIR827* gene altogether abolish miR827 production and confer resistance to *M. oryzae* infection. This resistance is accompanied by the reduction of leaf *Pi* content compared to wild-type plants, whereas Pi levels increase in the leaves of the blast-susceptible miR827 overexpressed or plants. In wild-type plants, miR827 accumulation in leaves decreases during the biotrophic phase of the infection process (Bundo et al., 2024). 58B was edited by CRISPR/Cas9, targeting a *Pi21* gene and effector-binding element (EBE) of the *OsSULTR3;6* gene, and the mutants 58b were obtained by *Agrobacterium*-mediated method, but the expression of defense-responsive genes was significantly upregulated after infection with rice blast (Yang et al., 2023). A simple single-guide RNA (sgRNA) was designed to create *oss5h1oss5h2oss5h3* triple mutants through CRISPR/Cas9-mediated gene mutagenesis. *oss5h1oss5h2oss5h3* exhibited stronger blast resistance in rice to *Xoo* than single *oss5h* mutants and also significantly upregulated *OsWRKY45* and *pathogenesis-related* (*PR*) genes in *OsS5H* gene editing (Liu et al., 2023). CRISPR/Cas9-mediated gene editing is employed to rapidly install mutations in three known broad-spectrum blast-resistant genes, *Bsr-d1*, *Pi21*, and *ERF922*, in an *indica* thermosensitive genic male sterile (TGMS) rice line Longke638S (LK638S) (Zhou et al., 2022). Hence, it infers that CRISPR-Cas9 technology emerges as a transformative tool for editing and the targeted modifications of blast resistance genes (*Pi* genes) and is also useful in gene knockout of susceptibility genes (*S* genes), stacking of resistant genes, which accelerates the development of blast-resistant rice varieties.

**Table 7:** Recent advances in techniques for improving blast resistance genes in rice.

Approaches	Gene transferred	Function of gene	References
Transgenic			
*Agrobacterium tumefaciens*-mediated transfer	*Pikh*	Blast resistance	Azizi et al., 2016
	*MoHrip1* and *MoHrip2*	Imparts resistance against blast and improvement in agronomic traits	Wang et al., 2017
Ac/Ds transposon vectors	*Pi21*	Blast resistance	Liang et al., 2020
Vector-mediated transformation	*Pib*, *Pi25*, and *Pi54*	Confers resistance against blast resistance	Peng et al., 2021
CRISPR Cas9, TALENS, RNAi, ZFNs			
Agrobacterium-mediated transformation of embryogenic calli with Cas9/gRNA expression binary vectors	*ERF922*	Transcription factors implicated in multiple stress responses	Wang et al., 2016
Induces plant defense responses for *Magnaporthe oryzae*	*OssSEC3A*	Interacts with *SNAP25*- type *t-SNARE* protein *OsSNAP32* which is responsible for blast resistance	Ma et al., 2018
CRISPR/Cas9	*Pi21*	Resistant to blast disease	Nawaz et al., 2020
Increases resistance by knocking out S gene or by causing mutation	S genes, *Pi21*, and *Bsr-d1*	Responsible for susceptible reaction in rice for blast	Tao et al., 2021
CRISPR/Cas9-mediated gene editing against blast resistance	*Bsr-d1*, *Pi21*, and *ERF922*	Improving blast resistances	Zhou Y et al., 2022
Zinc-finger domains	*OsiSAP8*	Resist drought and salinity	Krishnan et al., 2022
	*OsvWA1*, *OsvWA2*, *OsvWA6*, *OsvWA1 5*, *OsvWA16*, and *O svWA39*	Biotic stress resistance	Karkute et al., 2022
TALEN	*orf312*		Takatsuka et al., 2022
CRISPR/Cas9	*OsDjA2* and *OsERF104*	Rice blast resistance	Minmin et al., 2022
	*OsSULTR3*;*6*	Rice blast resistance	Yang et al., 2023
	*RNG1* or *RNG3*	Rice blast and bacterial blight resistance	Xu et al., 2023
	*OsHDT701*	Improves the resistance against blast	Mathsyaraja et al., 2024
Rice with knockdown *OsHDAC1* gene	*OsSSI2*, *OsF3H*, *OsRLR1*, and *OsRGA 5*	Enhanced broad-spectrum blast resistance	Hou et al., 2024

### Ectopic expression

The most notable advancement in varietal development for disease resistance is the use of genetic engineering to develop transgenic rice with enhanced disease resistance. This method is advantageous for introducing disease resistance into elite rice cultivars, as transgenic plants can acquire a single desired trait without altering the original genetic background. Several studies have been performed to confer the disease resistance in rice against *M. oryzae* (Peng et al., 2021; Li et al., 2021; Wang et al., 2017). Transgenic technology enables the precise manipulation of genes encoding the desired traits of interest by inserting foreign genes from unrelated species or silencing specific gene expression. *Agrobacterium* transformation and biolistic methods are the most commonly employed techniques for transferring a gene of interest into selected plant cells. *Agrobacterium*-mediated transformation ensures the stable integration of new genes into the targeted genome—for instance, enhanced resistance to blast fungus was achieved in rice by expressing genes such as rice chitinase [rice class-I chitinase gene, Cht-2 or Cht-3]. Coca et al. (2006) developed blast-resistant transgenic rice by transferring the *ER-CecA* gene from the giant silk moth *Hyalophora cecropia*. This gene was optimized to produce Cecropin A peptides in paddy, which is an are a member of antimicrobial protein families and which is a good indicator of the direct effect of a gene on the pathogen. Moreover, Wang et al. (2017) transferred *MoHrip1* and *MoHrip2* genes into rice through an *Agrobacterium tumefaciens*-based method used against blast resistance to produce the transgenic paddy plants and constrain the growth of fungal hyphae and also had a high water-retention capacity. Furthermore, marker-free transgenic rice was generated using maize’s Ac/Ds transposon vectors carrying fluorescent protein (GFP) and red fluorescent protein (mCherry) genetic markers to generate marker-free transgenic plants. *Pi21* gene was expressed in these transgenic plants to generate resistance against rice blasts. The transformed lines had good resistance against *M. oryzae* (Li et al., 2021).

Notably, three *Pi* genes, *viz*., *Pib*, *Pi25*, and *Pi54*, were transferred together into two rice varieties, the indica variety Kasalath and the japonica variety Zhenghan 10. The transformed varieties exhibited a good level of resistance against blast pathogens, but this gene pyramiding came with its baggage of linkage drag and pleiotropic effects of these genes. The transgenic plants were impairing many gene transcriptions, which ultimately interrupted the normal development of the plants (Peng et al., 2021). Glucan plays an important role in the growth and development of fungi, whereas glucanase can inhibit the growth of fungi by breaking glycosidic bonds and may be a promising target for developing rice varieties with broad-spectrum disease resistance. Researchers engineered a codon-optimized β-1,6-glucanase gene (*GluM*) derived from myxobacteria and introduced it into the japonica rice variety Zhonghua11 (ZH11). They generated numerous individual transgenic lines overexpressing *GluM*. From these, three single-copy, homozygous lines were selected at the T₃ generation for disease resistance analysis. The key outcomes compared to the non-transgenic ZH11 control are blast lesion area was reduced by ~82.71%, indicating a substantial enhancement against this major fungal disease in rice (Shen et al., 2023). Huang et al. (2023) revealed that transgenic plants overexpressing the *ATP2* gene were generated via genetic transformation in the Zhonghua11 (ZH11) genetic background, and the blast resistance and immune response of *ATP2*- overexpressing lines and wild-type plants were compared. When infected by the rice blast fungus, the transgenic rice plants exhibited stronger antioxidant enzyme activity and a greater ratio of chlorophyll a to chlorophyll b. The first cloning of a synthetic maize *chitinase* 1 gene and its insertion in rice cv. (Basmati 385) is *via Agrobacterium*-mediated transformation to confer resistance to the rice blast pathogen, *Pyricularia oryzae*. Transgenic lines were analyzed using molecular and functional techniques and confirmed by polymerase chain reaction with primer sets specific to *chitinase* and *hpt* genes. Furthermore, real-time PCR analysis of transformants indicated a strong association between transgene expression and elevated levels of resistance to rice blast in transgenic Basmati 385 plants (Anwaar et al., 2024). The potential future for transgenic crops can be used to generate new varieties or to create strong resistance barriers against blast disease.

The adoption of transgenic approaches for controlling rice blast disease, though promising, faces significant regulatory, ethical, and social challenges across countries due to their biosafety laws, public perception, and agricultural policies. Regulatory hurdles include stringent approval processes imposed by authorities such as America’s USDA (United States Department of Agriculture) and APHIS (Animal and Plant Health Inspection Service), Europe’s EFSA (European Food Safety Authority), and India’s GEAC (Genetic Engineering Appraisal Committee), which evaluate GM rice for potential environmental risks, allergenicity, and unintended gene flow to wild relatives (Singh et al., 2022). Ethical concerns arise over intellectual property rights, as transgenic rice varieties are often patented, raising fears of corporate monopolization and reduced access for smallholder farmers, particularly in Asia and Africa where rice is a staple food (Shukla et al., 2018). Additionally, public opposition to GM crops persists in regions like Europe and Japan, where consumers demand “GM-free” labels due to perceived health risks and environmental concerns despite scientific evidence supporting the safety of GM crops (Sabat and Tripathy, 2024). In contrast, China and the Philippines have begun approving GM rice, driven by food security needs and government-backed research (ISAAA, 2024). Conversely, India has maintained a strict stance against GM food crops, with only GM cotton approved. Simultaneously, public protests and concerns over corporate control hinder the approval of GM rice (Dutta et al., 2016). Developing rice varieties resistant to pests and diseases will help protect farmers from the adverse impacts of chemical insecticides and fungicides. Additionally, challenges posed by abiotic stressors like drought, extreme temperatures, and salinity, factors that hinder rice cultivation, can be mitigated by creating GM rice featuring genes that enhance tolerance to these stresses. Nonetheless, the commercialization of GM crops remains a divisive topic, as global acceptance continues to evolve (Dutta et al., 2016). Overall, the outlook for GM rice is encouraging; however, social acceptance remains a bottleneck, with misinformation and a lack of farmer awareness slowing the adoption rates (Sandhu et al., 2024). Thus, while transgenic rice holds immense potential for durable blast resistance, overcoming these regulatory, ethical, and social barriers will require transparent policies, farmer education, and region-specific regulatory frameworks that balance innovation with public trust.

Hence, it concluded that all approaches for blast-resistant rice varieties through genetic engineering and marker-assisted breeding have significantly progressed the field of rice cultivation. Nevertheless, the long-term stability of these resistance traits faces challenges due to the adaptive evolution of *Magnaporthe oryzae*, the organism responsible for rice blast disease. The pathogen’s considerable genetic variability allows it to overcome resistance provided by singular major resistance (R) genes, leading to the gradual breakdown of resistance in rice cultivars over time. For instance, the *Pi-ta* gene, which has demonstrated effectiveness against specific strains of *M. oryzae*, has been compromised due to mutations in the corresponding avirulence gene present in the pathogen, thus underscoring the coevolutionary dynamics between host resistance and pathogen virulence (Jia et al., 2016). To enhance the durability of blast resistance, strategies such as pyramiding multiple R genes and integrating quantitative trait loci (QTLs) associated with partial resistance have been adopted. This methodology aims to establish a broader and more sustainable defense against varied pathogen populations. Innovations in genomic tools, including CRISPR/Cas9-mediated gene editing, have expedited the precise stacking of resistance genes, thereby presenting a promising pathway for the development of rice varieties with enhanced and enduring blast resistance (Zhou et al., 2022). Ongoing surveillance of *M. oryzae* populations is essential to identify emerging virulent strains promptly. The integration of these strategies ensures the long-term stability of blast resistance traits in rice, consequently safeguarding global rice production against this widespread disease (Pedrozo et al., 2025).

## Future emerging diagnostic tools

### Nanotechnology

Nanotechnology has emerged as a promising avenue for the effective management of rice blast that poses a substantial threat to global rice production. Various nanosized materials, which include quantum dots, metallic nanoparticles, silica nanospheres, magnetic nanoparticles, silicon nanowires, carbon nanotubes, nanopores, graphene, nanostructured surfaces, and metal films, have exhibited immense potential in revolutionizing the development of diverse *in vitro* diagnostic assays for rice blast pathogens. These materials offer unique properties, such as a high surface area-to-volume ratio, tunable surface chemistry, enhanced sensitivity, and specific targeting capabilities, which are essential for the accurate detection and characterization of rice blast pathogens. By harnessing nanotechnology, diagnostic procedures that empower the effective management and mitigation of rice blast infection in global rice production are streamlined (Kashyap et al., 2017b). Utilizing nanotechnology principles, Yang et al. (2014) developed an electrochemical device for the early detection of blast fungal infection in rice, offering targeted delivery and reduced environmental impact. This nanodevice employed palladium nanoparticles (PdNPs) as catalysts within a 3,3′,5,5′-tetramethylbenzidine sulfate/H2O2 system, alongside immobilized mannose-binding jacalin-related lectin (*Osmbl*) from rice. Rhamnolipid (RL)-modified silica nanoparticles (SiO_2_NPs) based on excellent antimicrobial effectiveness against various phytopathogens, helping to reduce plant diseases (Li S. et al., 2024). We investigated the functions and mechanisms of RL@SiO_2_NPs in alleviating rice blast disease, resulting in a remarkable 10.80% decrease in disease incidence, 97.05% reduction in fungal growth, and 13.33% increase in shoot dry biomass, collectively lowering the infection pressure from the blast fungus. This treatment also enhanced the plants’ nutritional condition and bolstered their disease resistance by restoring nutrient balance and maintaining ion homeostasis. This is supported by a 23.84% increase in potassium levels in leaves, a 60.34% rise in silica concentration in roots, and reductions of 11.89% in magnesium and of 11.89% in iron levels in rice leaves. In summary, our results indicate that RL_0.1_@SiO_2_NPs enhance rice plant resistance to blast by boosting the *in vivo* antifungal activity, activating the antioxidant defense system, and improving nutrient absorption in rice seedlings. Shahid et al. (2025) reported that silver nanoparticles (AgNPs) act as a preventive measure to reduce rice blast in *Kocuria* species. *In vitro* antifungal activity showed that AgNPs significantly reduced the mycelial growth and conidial germination of *M. oryzae*. Their antifungal effects were more pronounced than those of propiconazole, a commonly used demethylation inhibitor fungicide for rice diseases. Similarly, zinc oxide nanoparticles (ZnO NPs) have been recognized as effective agents against *M. oryzae*, the causal agent of rice blast. They exhibit direct antifungal properties by inhibiting fungal conidiation and appressorium formation. Additionally, they enhance the basal resistance of rice plants by inducing the accumulation of reactive oxygen species and upregulating defense-related genes and also reduce abscisic acid levels, leading to increased stress tolerance in rice seedlings (Qiu et al., 2023). The synthesis of nickel–chitosan nanoparticles (Ni–Ch NPs) was carried out using nickel chloride, and its effectiveness in promoting plant growth and inhibiting *Pyricularia oryzae* (the blast pathogen) was evaluated. A significant increase in germination and growth characteristics, including shoot and root lengths as well as the number of lateral roots, was noted in paddy seeds treated with Ni–Ch NPs compared to the control group, highlighting an improved photosynthetic rate. Additionally, Asian rice exhibited reduced blast symptoms on leaves treated with NPs under glasshouse conditions, demonstrating 64% mycelial inhibition in Petri plates. These findings suggest that nickel–chitosan nanoparticles could act as an effective plant growth promoter in managing rice blast disease (Parthasarathy et al., 2023). Nano-particles derived from *Chaetomium* sp. extracts exhibited significant antifungal activity against *M. oryzae* (Song et al., 2018). These findings emphasize the potential of nanotechnology to create sustainable and efficient methods for managing rice blast, providing alternatives to traditional chemical treatments. Therefore, machine learning and artificial intelligence techniques are crucial for identifying and developing networks designed to detect blast disease in rice.

### Synthetic biology

Synthetic biology is a growing discipline which includes biology, genetic engineering, and computer science to design and construct new biological processes or perform specific functions (Benner and Sismour, 2005). This technique helps introgression or modify resistance (*R*) genes to improve the resistance against *Magnaporthe oryzae—*for instance, the *Pi54* gene has been identified and cloned from the *Indica* rice line Tetep, conferring resistance to blast disease. Researchers have developed DNA markers and isolated new alleles, such as *Pi54rh* and *Pi54* of, from wild rice species, contributing to the development of resistant rice varieties. The rice callus platform provides a unique opportunity to test strategies for the metabolic engineering of synthetic carotenoid pathways, leading to novel carotenoid-biofortified crops (Zhu et al., 2022). In transgenic rice plants, flowering time can be controlled by specific agrochemicals and yield-related traits like grain number, plant height, and heading date, which may lead to the production of crops suitable for growth in different climates and facilitate breeding for various agronomical traits by using synthetic biology (Okada et al., 2017). Hence, this perspective reveals the synthetic biology approach mostly used for the development of genetic engineering against traits and to explore the potential of protein construction to address the invasion and proliferation of *M. oryzae*, with the goal of identifying new drug targets and designing small-molecule compounds to manage this disease (Yan et al., 2024). This emerging technique is still not used for the development of resistance varieties against blast broad-spectrum disease.

### Machine learning and artificial intelligence

Machine learning and artificial intelligence have emerged as a powerful tool for managing blast disease caused by *Magnaporthe oryzae* (Rajpoot et al., 2023). Advances in automated and high-throughput imaging technologies have resulted in a deluge of high-resolution images and sensor data of plants. However, extracting patterns and features from this large corpus of data requires machine learning (ML) tools to enable data assimilation and feature identification for stress phenotyping (Gill et al., 2022). The four stages of the decision cycle in plant stress phenotyping and plant breeding activities where different ML approaches can be deployed are (i) identification, (ii) classification, (iii) quantification, and (iv) prediction (ICQP). We provide a comprehensive overview and user-friendly taxonomy of ML tools to enable the plant community to correctly and quickly apply the appropriate ML tools and best-practice guidelines for various biotic and abiotic stress traits (Singh et al., 2016). These technologies enable accurate disease detection, prediction, and decision-making by analyzing large datasets, including phenotypic, environmental, and genotypic information. Recently, the convolutional neural networks (CNNs) model has been used to differentiate healthy and infected leaves more efficiently and accurately. These AI models analyze multi-omics data to identify candidate genes and pathways involved in blast disease and utilize drones for mapping disease severity and are helpful for the amount of fungicide application against blast resistance in rice. Faster R-CNN, Cascade R-CNN, and YOLOv3 were used as the primary detection frameworks of deep learning methods used for spore identification against blast fungal disease in rice (Zhou H et al., 2024). The prediction of the severity of blast disease to classes 0, 1, 2, and 3, respectively, in rice was performed by using a linear SVM (support vector machine) machine (Varsha et al., 2024). Integration of a support vector machine classifier and convolutional neural networks is used to recognize and classify specific varieties of paddy plant diseases (Haridasan et al., 2023; Maheswaran et al., 2022). The visual patterns on the rice plants are processed using machine learning classifiers such as support vector machine (SVM), logistic regression, decision tree, naïve Bayes, random forest, linear discriminant analysis (LDA), and principal component analysis (PCA), and based on the classification results, plants are recognized as healthy or unhealthy for blast disease (Kumar et al., 2021). Deep learning and hyperspectral imaging methods, including the UeAMNet (Unsupervised Extraction Attention-based Mixed CNN) model, assess the efficacy of identifying infected and healthy cases of rice blast disease (Yin et al., 2025). Integrating UAV remote sensing with deep learning allows for efficient high-throughput field phenotyping in breeding for rice blast resistance (Zhang et al., 2025). Kaur and Sivia (2024) found that four deep learning convolutional neural networks (CNNs)—AlexNet, Inception, Xception, and Visual Geometry Group (VGG)—paired with machine learning models like decision tree (DT), random forest (RF), and support vector machine (SVM), are based on supervised learning in the automatic detection of leaf blast disease in rice. Automatic leaf identification of blast diseases is essential for several reasons, including minimizing yield loss, monitoring and forecasting infections, recognizing host resistance, and investigating fundamental host–pathogen interactions. Artificial neural networks (ANN) performance is enhanced by selecting parameters using the adaptive sunflower optimization (ASFO) algorithm. Subsequently, the infected area is delineated using a level set segmentation algorithm. Our evaluation of this method, based on accuracy, sensitivity, and specificity, indicates that the proposed technique achieves a maximum accuracy of 97.94% in predicting rice blast disease (Zheng et al., 2011). Lee et al. (2022) reported that machine learning methods utilize three artificial neural network (ANN)-based models to predict rice blast. These models incorporate two types of ANN—the feed-forward neural network (FFNN) and long short-term memory (LSTM)—using various input datasets for performance comparison. The Blast Weather FFNN model attained the highest recall score of 66.3% in rice blast prediction. The effectiveness of ANN-based disease prediction models improved by applying appropriate machine learning techniques and hyperparameter tuning to optimize input data. A machine learning algorithm facilitates the early detection of rice blast disease. For this system, images of both healthy rice leaves and those affected by the disease are collected. Features are extracted from the healthy parts and diseased areas of the rice leaves. The simulation results indicate an accuracy of 99% for images showing blast infection and 100% for healthy images in the training phase. In the testing phase, accuracy is recorded at 90% for infected images and 86% for healthy ones, respectively (Ramesh and Vydeki, 2018).

## Challenges

**Figure 5:**
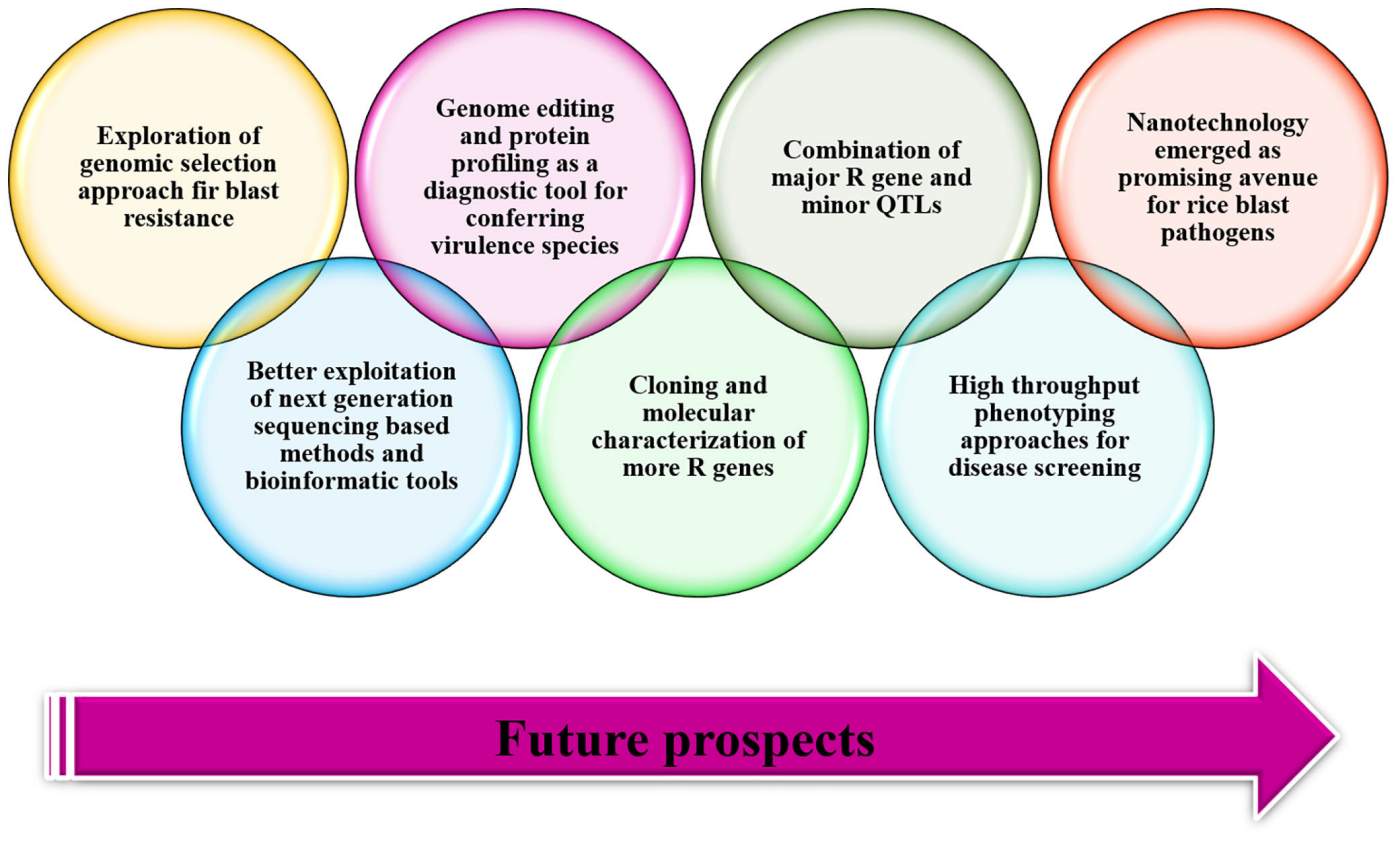
Future prospects for developing blast resistance in rice.

Blast disease in rice presents significant challenges due to its complex nature, geographical distribution, and the ever-changing characteristics of its pathogen. The fungus *Magnaporthe oryzae* is highly adaptable, showing rapid genetic variability that undermines the effectiveness of resistance in rice varieties. Differences in environmental conditions, such as humidity, temperature, and cropping systems across various regions, further complicate disease prediction and management strategies. Traditional management practices, including the use of fungicides, face issues related to environmental sustainability, the development of resistance in the pathogen, and high costs, particularly in resource-limited areas. Although biotechnological interventions show promise, they are hindered by the need for extensive research to identify durable resistance genes, the high costs associated with genetic engineering technologies, and regulatory challenges surrounding genetically modified organisms (GMOs) (**Figure 5**). Furthermore, breeding approaches require significant time investments, and integrating resistance genes often leads to trade-offs with yield or other agronomic traits. Many rice-growing regions also face limited access to high-throughput phenotyping, advanced diagnostic tools, and precise disease forecasting systems, which further hampers effective interventions. Socioeconomic factors, such as a lack of awareness among farmers, limited infrastructure, and difficulties in adopting advanced technologies, increase the challenges of achieving sustainable disease management on a global scale.

## Conclusions and future perspectives

Traditional control strategies prove ineffective at the commercial scale. Employing resistant varieties is the most efficient approach to mitigate disease occurrence and avoid pesticide-related risks. Through conventional plant breeding methods, numerous blast-resistant cultivars have been developed. However, molecular-level advancements in rice have opened new avenues for enhancing rice production systems. Molecular breeding techniques, including MAS, MABB, transgenic approaches, and genome editing, have effectively managed resistance. Furthermore, cloning *R* and *Avr* genes and studies of their products will deepen our understanding of host–pathogen interactions. Rice varieties carrying a single R gene for a specific pathogen race often lose their resistance over time due to the appearance of new virulent strains.

Understanding the genetic identity of the new *M. oryzae* race is important for the accurate employment of rice cultivars with different R genes. Stacking of multiple R genes will provide long-lasting resistance. In rice breeding programs against blast disease, various combinations of resistance R genes in a single host plant should be considered. Incorporating multiple race-specific R genes into elite rice cultivars is widely recognized as the most effective strategy for developing broad-spectrum and durable resistance to blast disease. Nevertheless, this approach may inadvertently foster the evolution of new pathogen races, potentially including super races that could overcome multiple major R genes and trigger severe epidemics. As a result, it is imperative to strategically employ race-specific R genes in breeding programs to preserve rice cultivars’ blast resistance, which is still poorly understood. To achieve broad-spectrum and long-lasting resistance, a strategic blend of significant R genes and minor QTLs is essential, leveraging conventional integrated breeding techniques, state-of-the-art genomic approaches, and gene editing tools.

Marker-assisted foreground and background selection can accelerate the development of near-isogenic lines (NILs). More expertise could be involved in performing recently developed transgenic and genome editing technologies that could enhance their application in creating blast-resistant rice through precise genetic modifications. Some recent techniques, such as allele mining, association mapping, and genome editing, will also play a vital role in controlling blast disease. Emerging technologies such as nanotechnology, machine learning, and artificial intelligence offer promising advancements that can help reduce blast disease in rice. Nanotechnology approaches encompass nanoformulated pesticides, nanosensors for early disease detection, and enhanced resistance mechanisms. These methods provide precise and eco-friendly strategies for managing diseases and improving plant immunity through nano-based formulations. Machine learning enables early prediction and precise disease identification by assessing environmental and genetic factors. Artificial intelligence improves disease management by refining control strategies, advancing resistance breeding, and supporting decision-making through predictive modeling. By integrating these cutting-edge technologies, rice farmers can manage diseases proactively, minimize chemical use, and boost crop yields. Future studies should focus on merging nanomaterials with AI-powered smart sensors to develop real-time, field-ready diagnostic tools. Moreover, deep learning models utilizing extensive field data can enhance disease prediction and refine disease management strategies.

However, due to the variable nature of pathogens, the need for regular research on the advancement of sustainable resistant cultivars will always be a never-ending process due to the co-evolution of pathogens. Future investigations should identify such S genes in rice to harness them through genome modification techniques for the development of blast-resistant varieties. These advanced diagnostic techniques are useful for the early detection of blast disease, which is helpful for prevention and effective strategies against the development of broad-spectrum durable blast resistance in rice.
